# Synergistic Protection by Isoquercitrin and Quercetin against Glutamate-Induced Oxidative Cell Death in HT22 Cells via Activating Nrf2 and HO-1 Signaling Pathway: Neuroprotective Principles and Mechanisms of *Dendropanax morbifera* Leaves

**DOI:** 10.3390/antiox10040554

**Published:** 2021-04-02

**Authors:** Hye-Jin Park, Ha-Neul Kim, Chul Young Kim, Min-Duk Seo, Seung-Hoon Baek

**Affiliations:** 1 College of Pharmacy and Research Institute of Pharmaceutical Science and Technology (RIPST), Ajou University, Suwon 16499, Korea; hyejin133@ajou.ac.kr (H.-J.P.); skylive02@ajou.ac.kr (H.-N.K.); 2 Department of Molecular Science and Technology, Ajou University, Suwon 16499, Korea; 3 College of Pharmacy and Institute of Pharmaceutical Science and Technology, Hanyang University, Ansan-si 15588, Korea; chulykim@hanyang.ac.kr

**Keywords:** glutamate-induced oxidative cell death, *Dendropanax morbifera* leaves, isoquercitrin, quercetin, nuclear factor erythroid 2-related factor 2, heme oxygenase-1, synergism

## Abstract

*Dendropanax morbifera* leaves (DML) have long been used as traditional medicine to treat diverse symptoms in Korea. Ethyl acetate-soluble extracts of DML (DMLE) rescued HT22 mouse hippocampal neuronal cells from glutamate (Glu)-induced oxidative cell death; however, the protective compounds and mechanisms remain unknown. Here, we aimed to identify the neuroprotective ingredients and mechanisms of DMLE in the Glu-HT22 cell model. Five antioxidant compounds were isolated from DMLE and characterized as chlorogenic acid, hyperoside, isoquercitrin, quercetin, and rutin by spectroscopic methods. Isoquercitrin and quercetin significantly inhibited Glu-induced oxidative cell death by restoring intracellular reactive oxygen species (ROS) levels and mitochondrial superoxide generation, Ca^2+^ dysregulation, mitochondrial dysfunction, and nuclear translocation of apoptosis-inducing factor. These two compounds significantly increased the expression levels of nuclear factor erythroid-2-related factor 2 (Nrf2) and heme oxygenase 1 (HO-1) in the presence or absence of Glu treatment. Combinatorial treatment of the five compounds based on the equivalent concentrations in DMLE showed that significant protection was found only in the cells cotreated with isoquercitrin and quercetin, both of whom showed prominent synergism, as assessed by drug–drug interaction analysis. These findings suggest that isoquercitrin and quercetin are the active principles representing the protective effects of DMLE, and these effects were mediated by the Nrf2/HO-1 pathway.

## 1. Introduction

Glutamate (Glu)-induced oxidative cell death has been implicated in diverse neurological disorders, such as stroke, traumatic brain injury, epilepsy, and neurodegenerative diseases [1,2,3,4]. The pathological stimuli on the central nervous system induce the accumulation of extracellular Glu in glutamatergic synapses, thereby killing neuronal cells via excitotoxicity [5]. Glu receptors (GluRs) and cystine/Glu antiporters (system $x_{c}^{-}$) are the major responders that initiate excitotoxicity [6]. Excessive extracellular Glu activates ionotropic and metabotropic GluRs, resulting in the massive influx of extracellular Ca^2+^ and cytosolic release of Ca^2+^ from the endoplasmic reticulum (ER). It also reverses the action of $x_{c}^{-}$ to facilitate the export of cystine and import of Glu and leads to the inhibition of glutathione (GSH) synthesis. Imbalance of intracellular Ca^2+^ and GSH levels ultimately accelerates oxidative stress, mitochondrial dysfunction, and neuronal cell death. The mechanism of Glu-induced cytotoxicity depends on the cell type. HT22 mouse hippocampal neuronal cells that are rich in $x_{c}^{-}$ and lacking GluRs are representative cell lines used to study Glu-induced oxidative cell death, which is termed oxytosis [6,7,8].

The Kelch-like erythroid cell-derived protein with cap-n-collar homology (ECH)associating protein (Keap1)/nuclear factor erythroid-2-related factor 2 (Nrf2) pathway is an evolutionarily conserved antioxidant defense mechanism that protects against oxidative and xenobiotic stress [9,10]. Under normal conditions, Keap1 maintains low levels of cytosolic Nrf2 levels through the formation of a complex with Nrf2, resulting in its ubiquitination-mediated proteasomal degradation. Under oxidative stress or xenobiotic challenge, electrophilic species modify the structural conformation of Keap1, leading to the release of free Nrf2, slowdown of degradation, and accelerated nuclear Nrf2 accumulation. Nrf2 binding to the antioxidant response element (ARE) augments the expression of several target genes involved in detoxification, antioxidant, anti-inflammatory, and metabolic processes [11,12]. As the Keap1/Nrf2 axis has emerged as a promising therapeutic target, diverse chemical modulators or drug candidates have been developed or are under clinical trials [13,14]. Of note, natural products and dietary phytochemicals are known to modulate the Keap1/Nrf2 pathway and contribute to the maintenance of redox homeostasis in neuronal cells and nervous systems that are vulnerable to oxidative stress [15,16]. Since natural products have long been used as foods or herbal medicines, they are promising resources for the discovery of safe and effective modulators of Keap1/Nrf2.

*Dendropanax morbifera* Léveille (DM, Araliaceae) is an endemic species in Korea. Leaves and other parts of DM have been used as a cure for the treatment of skin diseases, infectious diseases, headaches, and other symptoms [17]. Diverse phytochemicals and pharmacological efficacies of DM have been summarized in a recently published review article [18]. Hundreds of chemicals, including phenolic compounds, polyacetylenes, flavonoids, tannins, essential oils, terpenoids, and alkaloids have been isolated and identified in DM. DM extracts or its components are responsible for broad pharmacological activities, including antioxidant, anti-cancer, anti-diabetic, anti-inflammatory, and protective effects on organs. Of note, along with the increase in the aging population, there has been growing attention on the neuroprotective activities of DM. DM extracts improved cognitive dysfunction or memory loss induced by heavy metal exposure, hypothyroidism, and diabetes in rats [19,20,21,22]. Oxidative stress has emerged as a cause of many nervous system diseases, such as neurodegenerative diseases, traumatic brain injury, stroke, and aging [23,24,25]. The antioxidant effects of DM extracts underlie their neuroprotective activities, but the bioactive constituents responsible for neuroprotection remain unclear.

In our previous study, we demonstrated that ethyl acetate-soluble extracts of DM leaves (DMLE) rescued HT22 cells from Glu-induced oxidative cell death via inhibition of oxidative stress, mitochondrial dysfunction, and apoptosis-inducing factor (AIF)-dependent apoptotic cell death [17]. The aim of this study was to elucidate the neuroprotective mechanisms of DM leaves and their mechanisms of action.

## 2. Materials and Methods

### 2.1. Chemicals and Reagents

L-Glutamic acid monosodium salt hydrate (Glu), *N*-acetyl-L-cysteine (NAC), L-ascorbic acid, chlorogenic acid, rutin hydrate, quercetin 3-D-galactoside (hyperoside), quercetin 3-β-D-glucoside (isoquercitrin), quercetin, 3-(4,5-dimethylthiazol-2-yl)-2,5-diphenyl tetrazolium bromide (MTT), tetramethyl rhodamine ethyl ester (TMRE), 5,5′,6,6′-tetrachloro-1,1′3,3′-tetraethylbenzamidazol-carboncyanine (JC-1), 2,2-diphenyl-1-picrylhydrazyl (DPPH), and 4-(2-hydroxyethyl)piperazine-1-ethanesulfonic acid (HEPES) were purchased from Sigma-Aldrich (St. Louis, MO, USA). Hoechst 33342, propidium iodide (PI), 2′,7′-dichlorodihydrofluorescein diacetate chloromethyl derivative (CM-H_2_DCFDA), MitoSox Red, Fluo-4 AM, phosphate-buffered saline (PBS), and Hanks’ balanced salt solution (HBSS) were purchased from Invitrogen (Thermo Fisher Scientific, Waltham, MA, USA). Fluorescein isothiocyanate (FITC)-conjugated Annexin V/PI assay (BD Pharmingen Annexin V FITC apoptosis detection kit) was purchased from BD Biosciences (San Jose, CA, USA). High-performance liquid chromatography (HPLC)-grade water and acetonitrile were purchased from JT Baker (Avantor, PA, USA). The primary antibodies and horseradish peroxidase (HRP)-conjugated secondary antibodies were purchased from Cell Signaling Technology (Danvers, MA, USA; Nrf2, #12721S; heme oxygenase 1, HO-1, #43966S; LC3A/B, #4108S; AIF, #5318S; superoxide dismutase 2, SOD2, #13141S; HRP-anti-rabbit IgG, #7074), Novelty Nobility Inc. (Suwon, Korea; α-tubulin, #CPI-101), and Santa Cruz Biotechnology (Santa Cruz, CA, USA; Lamin B1, #SC-6216; Keap1, #SC-15246; HRP-anti-goat IgG, #SC-2020; HRP-anti-mouse IgG, #SC-2020).

### 2.2. Plant Material

Dried DM leaves (DML) were purchased from Hwangchill Korea Corporation (Gangjin-gun, Jeollanam-do, Korea). It was the same batch with a voucher specimen (A16025) deposited at the pharmacognosy laboratory in our institute and authenticated using DNA barcoding assay in our previous study [17].

### 2.3. Extraction, Isolation, and Characterization of Compounds

Extraction and fractionation were carried out following the method described in our previous study [17]. Briefly, the dried DML (200 g) was refluxed with 70% methanol for 3 h, and extraction was repeated for three times. After extracts were pooled and evaporated, the dried extract was resuspended in water. Solvent fractions were prepared using consecutive liquid–liquid extraction with *n*-hexane, chloroform, ethyl acetate, and *n*-butanol. Each fraction was evaporated to dryness and stored in a desiccator until use. The ethyl acetate-soluble fraction was subjected to open column chromatography (4 × 45 cm) using silica gel and a solvent mixture of ethyl acetate/methanol (50:1–1:1, *v*/*v*). Five compounds were isolated from the sub-fractions using preparative HPLC. The structure was identified using nuclear magnetic resonance (NMR; Varian Mercury 400, Varian, Palo Alto, CA, USA; Bruker Avance 500, Bruker Avance 700, Bruker, Billerica, MA, USA) and LC-ESI-MS (Micromass ZQ, Waters, Milford, MA, USA).

### 2.4. Antioxidant Activity Assays

The antioxidant activities of the isolated compounds were assessed using DPPH radical scavenging, superoxide radical scavenging, and reducing power activity assays, according to the optimized methods in our previous study [17]. Ascorbic acid was used as the positive control.

#### 2.4.1. DPPH Radical Scavenging Assay

The sample and DPPH solutions were prepared in methanol and assay was performed in a 96-well plate. After mixing the sample solution (2–200 µM, 80 µL) and DPPH solution (0.1 mM, 80 µL), reaction mixtures were incubated for 20 min at room temperature (RT) in the dark. The absorbance at 517 nm was measured using a microplate reader (Biotek Instruments Inc., Winooski, VT, USA). Methanol was used as the control. Activity (%) was calculated using the following equation:

DPPH radical scavenging activity (%) = (1 − *A_S_*/*A_C_*) × 100 (1)

where *A_C_* is the absorbance of the control and *A_S_* is the absorbance of the sample.

#### 2.4.2. Superoxide Radical Scavenging Assay

Samples were dissolved in dimethyl sulfoxide (DMSO; Sigma-Aldrich) to make 50 mM. This was diluted with water to prepare sample solution (2–200 µM). All reagent solutions including β-nicotinamide adenine dinucleotide (NADH, 500 μM), nitrotetrazolium blue chloride (NBT, 300 μM), and phenazine methosulfate (PMS, 60 μM) were prepared in 0.2 M phosphate buffer (pH 7.4). For assay, reaction mixture was prepared by mixing the sample solution (2–200 µM, 90 µL), NADH (500 μM, 30 μL), NBT (300 μM, 30 μL), and PMS (60 μM, 30 μL) in the wells of a 96-well plate. After reaction (5 min, RT, dark), the absorbance was measured at 560 nm. Water was used as control. Activity (%) was calculated using the following equation:

Superoxide radical scavenging activity (%) = [1 − (*A_S_* − *A_SB_*)/(*A_C_* − *A_CB_*)] × 100 (2)

where *A_S_* is the absorbance of the sample, *A_SB_* is the absorbance of the sample blank (without NADH), *A_C_* is the absorbance of the control, and *A_CB_* is the absorbance of the control blank (without NADH).

#### 2.4.3. Reducing Power Assay

Sample solution (0.5 mL; 16–1600 μM in methanol) was mixed with phosphate buffer (2.5 mL; 0.2 M, pH 6.6) and potassium ferricyanide (2.5 mL; 1%, *w*/*v*). The mixtures were incubated at 50 °C for 20 min. After incubation, trichloroacetic acid (2.5 mL; 10%, *w*/*v*) was added to the mixture, which was then centrifuged at 3000× *g* for 10 min. The supernatant (2 mL) was mixed with water (2 mL) and FeCl_3_ solution (0.4 mL; 0.1%, *w*/*v*), followed by incubation for 2 min at RT to produce the Prussian blue. The absorbance was measured at 700 nm. Various concentrations of ascorbic acid (positive control) were tested until they reached an absorbance plateau at 700 nm. Activity (%) was calculated using the following equation:

Reducing power activity (%) = [*A_S_*/*A_PC(plateau)_*] × 100 (3)

where *A_S_* is the absorbance of the sample and *A_PC(plateau)_* is the maximal absorbance of positive control at the plateau.

### 2.5. Cell Culture and Treatments

The HT22 cell line, a mouse hippocampal neuronal cell line, was kindly given by Dr. Pae, Ae Nim, Korea Institute of Science and Technology (Seoul, Korea). Cells were cultured in Dulbecco’s modified Eagle’s medium (DMEM; Gibco, Thermo Fisher Scientific) supplemented with 10% fetal bovine serum (FBS; HyClone, Logan, UT, USA) and antibiotics (100 U/mL penicillin and 100 µg/mL streptomycin, Gibco) at 37 °C in humidified 5% CO_2_ incubator. Cells were seeded in multi-well plates or confocal culture dishes at a density of 15,000 cells/cm^2^ and incubated for 24 h, followed by treatment. The Glu of 4 mM was used for inducting the oxidative cell death. Various concentrations of samples were treated for indicated incubation times. Sample stock solutions were prepared in DMSO. The DMSO concentration was not more than 0.2% in the treatment.

### 2.6. Cell Viability Assay

Cell viability was measured using MTT assay. After the addition of MTT solution (0.5 mg/mL), cells were incubated at 37 °C for 3 h. After removing the media, 100 µL of DMSO was added to each well to dissolve the formazan crystals. Absorbance was measured at 540 nm using a microplate reader (Biotek Instruments Inc., Winooski, VT, USA). Cell viability was expressed as a percentage of untreated normal cells.

### 2.7. PI/Annexin V Apoptosis Analysis

Apoptosis was analyzed by flow cytometry using the FITC–Annexin V apoptosis detection kit (BD Biosciences, San Diego, CA, USA) following the manufacturer’s instructions. Cells in a 6-well plate were treated with drugs for 8 h, followed by scraping. The cell suspension in binding buffer were stained with PI and FITC-conjugated Annexin V for 15 min at RT in the dark, followed by analysis using flow cytometry (BD FACSCanto II, BD Pharmingen, San Jose, CA, USA). In the Annexin V/PI dot plots of normal cells, quadrants were assigned to distinguish the live (Annexin V^−^/PI^−^), early apoptotic (Annexin V^+^/PI^−^), late apoptotic (Annexin V^+^/PI^+^), and necrotic cells (Annexin V^−^/PI^+^). The cell population (%) of live or cell death in drug treated cells were determined based on the assigned quadrants. Flow cytometry data were analyzed using FlowJo software (version10, Ashland, OR, USA).

### 2.8. Measurement of Intracellular and Mitochondrial ROS Generation

Generation of intracellular and mitochondrial ROS was assessed using CM-H_2_DCFDA and MitoSox Red, respectively. Labeling the cells was performed following the manufacturer’s instructions. The buffers used for labeling procedures were PBS for CM-H_2_DCFDA and HBSS for MitoSox Red. The harvested cells were washed twice with washing buffer and stained with CM-H_2_DCFDA (5 µM in PBS) for 15 min or MitoSox Red (5 µM in HBSS) for 10 min at 37 °C in the dark, followed by washing thrice. The cellular DCF or oxidized MitoSox Red were analyzed using flow cytometry. In the histogram (x-axis, fluorescence intensity; y-axis, cell population) of normal cells, positive cells, and negative cells were distinguished by a marker on the x-axis. Intracellular and mitochondrial ROS generation was expressed as positive cells (%), as determined by the same marker.

### 2.9. Measurement of Intracellular Calcium Levels

Intracellular Ca^2+^ levels were evaluated using the fluorescent Ca^2+^ indicator Fluo-4 AM, according to the manufacturer’s instructions. After treatment, cells in a culture plate were incubated with Fluo-4 AM (2 µM in HBSS) for 30 min at 37 °C in the dark. After washing twice with HBSS, the labeled cells were visualized using a fluorescence microscope (Eclipse Ti-U, Nikon, Tokyo, Japan). Intracellular Ca^2+^ levels were expressed as a percentage of Fluo-4 positive cell count relative to the total cell count.

### 2.10. Measurement of Mitochondrial Membrane Potential

Mitochondrial membrane potential was determined using fluorescence probes (JC-1 and TMRE) following the method described in our previous paper [17]. The harvested cells were stained with JC-1 (5 μM) or TMRE (200 nM) for 15 min at 37 °C in the dark. Stained cells were washed twice with PBS, followed by flow cytometry analysis. The filter (488 nm) was used for excitation of JC-1. JC-1 aggregates (red, polarized mitochondria) and JC-1 monomers (green, depolarized) were analyzed using 595 nm and 525 nm emission filters, respectively. For TMRE-labeled cells, 544 nm excitation and 590 nm emission filters were used, respectively. Mitochondrial membrane potential was expressed as a percentage of JC-1 fluorescence ratio (red/green) or TMRE fluorescence intensity relative to normal cells.

### 2.11. Measurement of Mitochondrial Membrane Permeabilization

Mitochondrial membrane permeabilization was evaluated using the Image-iT™ LIVE mitochondrial permeability transition pore assay kit (Invitrogen) following the manufacturer’s protocol. Cells in a confocal culture dish were stained with warm labeling solution (1 μM calcein-AM, 200 nM MitoTracker Red, 10 μg/mL Hoechst 33342, 1 mM CoCl_2_, 10 mM HEPES, 2 mM L-glutamine, and 100 μM succinate in HBSS) for 15 min at 37 °C in the dark. Ionomycin (1 μM, positive control) was treated to cells for 10 min before labeling. After washing twice with HBSS, stained cells were visualized using a confocal microscope (C2 Plus; Nikon, Tokyo, Japan). Mitochondrial permeability transition was expressed as a percentage value of mean calcein fluorescence intensity in cells relative to normal cells.

### 2.12. Subcellular Fractionation

Subcellular fractionation was performed using the NE-PER Nuclear and Cytoplasmic Extraction Reagent Kit (Thermo Fisher Scientific) according to the manufacturer’s instructions. Cells were harvested by scraping, followed by lysis in cytoplasmic extraction buffer containing protease inhibitors for 10 min on ice. After centrifugation at 16,000× *g* for 5 min, the supernatant (cytoplasmic fraction) was recovered. Then, pellet was further lysed in nuclear extraction buffer for 40 min on ice. After centrifugation at 16,000× *g* for 10 min, the supernatant (nuclear fraction) was recovered. Cytoplasmic and nuclear fractions were used for western blotting analysis.

### 2.13. Western Blotting

Protein concentration in the lysates was determined using the Bradford assay with bovine serum albumin as a reference standard (Pierce™ Coomassie Bradford Protein Assay Kit, Thermo Fisher Scientific). Samples containing equal protein amounts in sample buffer were loaded on 4–15% SDS-PAGE precast gels (Bio-Rad, Hercules, CA, USA) and separated by electrophoresis. After proteins were transferred onto polyvinylidene fluoride membrane, the membranes were blocked with blocking buffer (Tris-buffered saline with 5% non-fat dry milk and 0.01% Tween-20) for 1 h at RT. Then, the membranes were incubated with each primary antibody solution (1:1000 in blocking buffer) at 4 °C overnight. The membranes were washed with Tris-buffered saline with 0.01% Tween-20 and incubated with horseradish peroxidase-conjugated secondary antibodies (1:5000 in blocking buffer). Protein signals were visualized using an enhanced chemiluminescence detection reagent. Protein band images were obtained using an LAS 500 image system (GE Healthcare, Chicago, IL, USA). Blot signals were quantitated by densitometry analysis using ImageJ software (NIH, Bethesda, MD, USA). Protein levels were normalized to those of the corresponding loading controls.

### 2.14. Molecular Docking Simulation

Ligand docking simulations between the isolated compounds and the BTB domain of Keap1 (Keap1-BTB; PDB entry: 4CXT) or mutant BTB domain at C151W of Keap1 (Keap1-BTB-C151W; PDB entry: 4CXJ) were performed using Autodock vina software [26]. Following the addition of polar hydrogens and Gasteiger charges to ligands and receptors, the conformational energies of the Keap1 receptor and ligands were minimized by 100 steepest descent steps using UCSF Chimera [27]. For the analysis of grid-based molecular docking, the center position of the grid box for docking was selected based on the information of the bound ligand. The docked conformations between Keap1-BTB and the compounds were ranked as per the predicted binding free energy (affinity) values given in kcal/mol. Docking results of all compounds with Keap1-BTB were visualized using UCSF Chimera.

### 2.15. Quantitative HPLC-UV Assay and Method Validation

HPLC-UV assays were performed to quantitate chlorogenic acid, rutin, hyperoside, isoquercitrin, and quercetin in DMLE and DMLB (*n*-butanol soluble extracts of DML). Five levels of standard mix solution (1–10 μg/mL for chlorogenic acid, rutin, hyperoside, and isoquercitrin; 0.1–1 μg/mL for quercetin) were prepared in methanol and used as calibrators. Sample solutions of DMLE (50 μg/mL) and DMLB (20 μg/mL) were prepared by dissolving the dried extracts with methanol. After filtration (0.22 μm pore size), calibrators and samples were injected to HPLC system equipped with a photodiode array detector (1260 infinity, Agilent Technologies, Santa Clara, CA, USA). Chromatographic separation was performed using a Hypersil GOLD-C18 column (5 μm, 250 mm × 4.6 mm i.d.; Thermo Fisher Scientific, San Jose, CA, USA) with a Security guard column (Phenomenex, Torrance, CA, USA). The mobile phase consisted of solvent A (0.1% formic acid in water, *v*/*v*, pH 2.7) and solvent B (acetonitrile) with the following elution profile: 0 min 8% B, 4 min 15% B, 8 min 16% B, 20 min 16% B, 40 min 40% B. The mobile phase flow rate was 0.8 mL/min. The injection volume was 5 μL, and the UV wavelength was 254 nm. The peaks were identified by comparing the retention times and UV spectra of the standard and sample. The proposed HPLC-UV method was validated in terms of specificity, linearity, limit of detection (LOD), limit of quantification (LOQ), precision, and accuracy according to the International Conference on Harmonization (ICH) guidelines [28]. Specificity was determined using the identification method described above. Linearity in specified concentration ranges of the analyte was assessed using the correlation coefficient (R^2^) in the calibration curves. The signal-to-noise (S/N) ratio method was used to calculate the LOD (concentration of analyte in S/N = 3) and LOQ (concentration of analyte in S/N = 10). Replicates of standard spiked samples at three concentration levels covering the concentration range were used to evaluate the accuracy (% recovery) and precision (% relative standard deviation). A method validation study was carried out for three consecutive days to assess the intermediate precision.

### 2.16. Analysis of Synergistic Effect

Drug–drug interactions between isoquercitrin and quercetin were studied using the Chou–Talalay method [29,30]. Stock solutions of isoquercitrin and quercetin combination were prepared in DMSO at various molar concentration ratios (quercetin: isoquercitrin = 1:5, 1:10, 1:20). The mixed solutions were serially diluted with medium and treated with 4 mM Glu for 24 h. Compound alone was used as a control. Cell viability was measured using MTT assay. The inhibitory activity of the sample was calculated using the following equation:

Inhibitory activity (%) = [(*A_S_* − *A_C_*)/(*A_N_* − *A_C_*)] × 100

where *A_S_* is the absorbance of the sample and Glu, *A_C_* is the absorbance of the Glu alone (control), and *A_N_* is the absorbance of the untreated (normal). Data consisting of concentrations and inhibitory activities were analyzed using CompuSyn software (ComboSyn, Inc., Paramus, NJ, USA). In the dose–effect curve, the dose was the concentration of the samples and the effect was the inhibitory activity. Isobologram, combination index (CI), and dose-reduction index (DRI) were calculated at diverse effect levels (Fa, fraction affected; inhibitory activity). Drug–drug interactions were finally determined based on the CI value, that is, CI < 1, synergism; CI = 1, additivity; CI > 1, antagonism.

### 2.17. Statistical Analysis

All data obtained from at least three independent experiments were expressed as mean ± standard error of the mean unless otherwise specified. Statistical analysis was performed using SPSS (version 22.0; SPSS Inc., Chicago, IL, USA). Significant differences were determined by Student’s *t*-test for between the groups or One-way ANOVA followed by Tukey’s multiple comparison test for among the groups.

## 3. Results

### 3.1. Extraction, Isolation, and Structual Characterization of Antioxidant Compounds from DML

**Figure 1:**
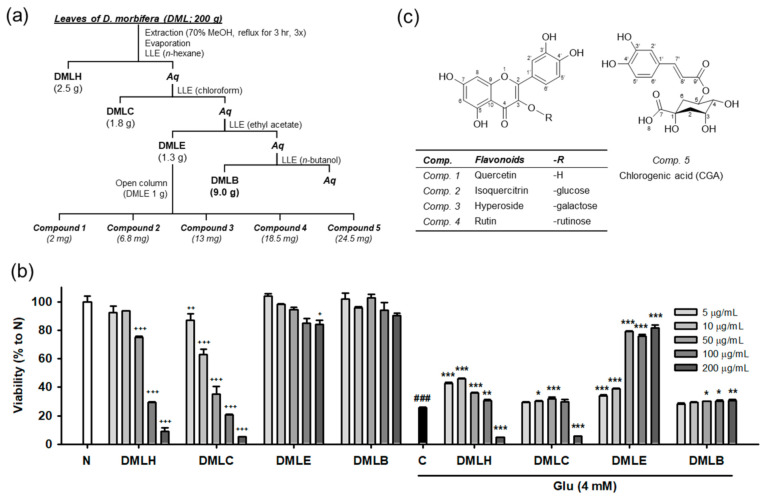
Isolation and structure of compounds from the *Dendropanax morbifera* leaves. (**a**) Scheme of fractionation and isolation; (**b**) Cytotoxicity and protective effect of DML extracts on Glu-induced HT22 cell death; (**c**) Structure of isolated compounds; Student’s *t*-test (N vs. C, ^###^ *p* < 0.001); One-way ANOVA (N vs. drugs, ^+^ *p* < 0.05, ^++^ *p* < 0.01, ^+++^ *p* < 0.001; C vs. Glu + drugs, * *p* < 0.05, ** *p* < 0.01, *** *p* < 0.001); DMLH, hexane extract; DMLC, chloroform extract; DMLE, ethyl acetate extract; DMLB, *n*-butanol extract; *Aq*, aqueous fraction; LLE, liquid–liquid extraction; Glu, glutamate; N, non-treated normal cells; C, control cells treated with Glu alone.

Our previous study demonstrated that DMLE significantly protects HT22 neuronal cells against Glu-induced cell death [17]. Oxidative stress plays a lethal role in Glu-induced excitotoxicity in neurological disorders [1,2,6]. The active compounds in DMLE are expected to have potent antioxidant activities. To identify the active compounds, extraction and isolation using chromatographic techniques were carried out, as shown in Figure 1a. DM leaves that were previously authenticated using DNA barcoding analysis were extracted and fractionated into four solvent extracts: *n*-hexane extract (DMLH), chloroform extract (DMLC), DMLE, and *n*-butanol extract (DMLB). Consistent with our previous data [17], DMLE was an active fraction that significantly rescued HT22 cells from Glu toxicity (Figure 1b). Five compounds were isolated from DMLE. Their spectral data, such as NMR and mass spectra, were measured (Figures S1–S10 and Table S1). These compounds were characterized as quercetin (comp. 1; [31]), isoquercitrin (comp. 2; [32]), hyperoside (comp. 3; [33]), rutin (comp. 4; [33]), and chlorogenic acid (comp. 5; [34]). These are natural polyphenol compounds such as flavonols, flavonol glycosides, and phenolic acids. The others have already been reported in DM, but to our knowledge, this is the first report of the extraction and identification of isoquercitrin and hyperoside in DM [18].

### 3.2. Antioxidant Activities of Isolated Compounds

**Figure 2:**
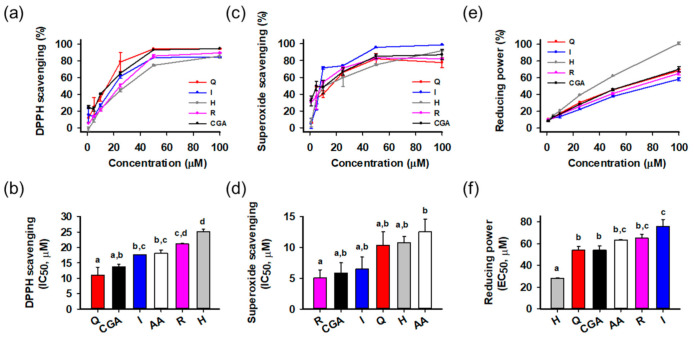
Antioxidant activities of isolated compounds. (**a**,**b**) 2,2-Diphenyl-1-picrylhydrazyl (DPPH) radical scavenging activities and half maximal inhibitory concentration (IC_50_); (**c**,**d**) Superoxide radical scavenging activities and IC_50_; (**e**,**f**) Reducing power activities and half maximal effective concentration (EC_50_); Different lowercase letters above bars indicate significant differences among the groups (One-way ANOVA, *p* < 0.05); AA, ascorbic acid; CGA, chlorogenic acid; H, hyperoside; I, isoquercitrin; Q, quercetin; R, rutin.

The antioxidant activities of the isolated compounds were evaluated using radical scavenging and reducing power assays (Figure 2). Ascorbic acid was a positive control. Activities were compared on the basis of half maximal inhibitory or effective concentration (IC_50_ or EC_50_, respectively) calculated from the relationship between concentration and activity. All compounds showed potent radical scavenging and reducing power activities in a concentration-dependent manner. In the DPPH radical scavenging assay (Figure 2a,b), quercetin (IC_50_ = 11.0 ± 2.6 μM) showed the greatest activity than the others, followed by chlorogenic acid (13.8 ± 0.7 μM), isoquercitrin (17.6 ± 0.1 μM), ascorbic acid (18.2 ± 1.0 μM), rutin (21.1 ± 0.2 μM), and hyperoside (25.2 ± 0.8 μM). In the superoxide radical scavenging assay (Figure 2c,d), all compounds showed significant activities stronger than ascorbic acid, and rutin (IC_50_ = 5.0 ± 1.3 μM) was the most potent, followed by chlorogenic acid (5.8 ± 1.7 μM), isoquercitrin (6.5 ± 2.0 μM), quercetin (10.4 ± 2.2 μM), hyperoside (10.8 ± 1.0 μM), and ascorbic acid (12.6 ± 2.1 μM). In reducing power assays (Figure 2e,f), hyperoside (EC_50_ = 28.3 ± 0.2 μM) showed the greatest activities significantly different from the others. Statistical analysis indicated that all isolated compounds showed potent antioxidant activities in the cell-free assays. Flavonols and polyphenols are well-known antioxidant natural compounds, and isolated compounds also showed comparable or superior antioxidant effects to ascorbic acid. To assess the effect on cellular protection, we next examined the protective activities using a cell-based system.

### 3.3. Isoquercitrin and Quercetin Inhibit Glu-Induced Apoptotic Cell Death in HT22 Cells

**Figure 3:**
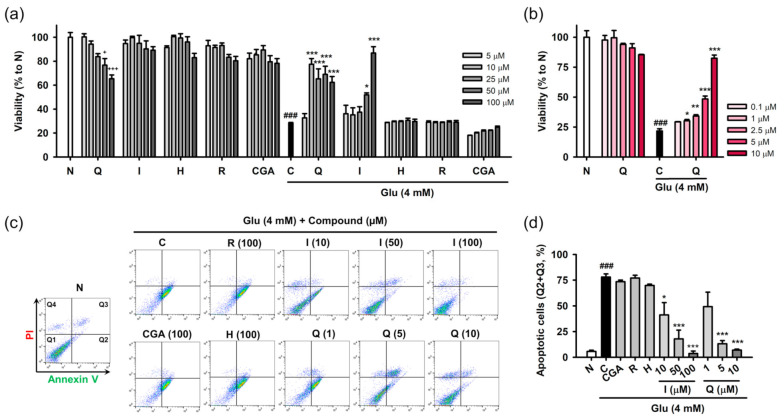
Effect of isolated compounds on the Glu-induced apoptotic cell death in HT22 cells. (**a**,**b**) Cytotoxicity and protective effect of isolated compounds on Glu-induced loss of cell viability. HT22 cells were treated with compound and/or Glu for 24 h. Cell viability was measured using MTT assay; (**c**,**d**) Effect of isolated compounds on Glu-induced apoptosis. HT22 cells were treated with compounds and/or Glu for 8 h. Apoptosis was measured using PI/Annexin V staining and flow cytometric analysis. Apoptotic cells (%) represents the sum of Annexin V-positive/PI-negative (Q2) and Annexin V-positive/PI-positive cell population (Q3); Student’s *t*-test (N vs. C, ^###^ *p* < 0.001); One-way ANOVA (N vs. drugs, ^+^ *p* < 0.05, ^+++^ *p* < 0.001; C vs. Glu + drugs, * *p* < 0.05, ** *p* < 0.01, *** *p* < 0.001); CGA, chlorogenic acid; H, hyperoside; I, isoquercitrin; Q, quercetin; R, rutin; Glu, glutamate; N, non-treated normal cell; C, control cells treated with Glu only; MTT, 3-(4,5-dimethylthiazol-2-yl)-2,5-diphenyl tetrazolium bromide; PI, propidium iodide.

Protective effects of the isolated compounds against Glu-induced cell death were assessed using cell viability and the PI/annexin V apoptosis assay. HT22 cells were treated with the compounds and/or 4 mM Glu for 24 h to assess their inherent cytotoxicity or protective effects on Glu toxicity (Figure 3a,b). Quercetin showed cytotoxicity in a concentration-dependent manner, and significant cytotoxicity was found at 50 μM. The other compounds were not toxic to HT22 cells at the test concentration (5–100 μM). Glu (4 mM) significantly reduced the viability of HT22 cells. Quercetin and isoquercitrin significantly restored the Glu-induced loss of HT22 cell viability, whereas the others failed to rescue the cells. As the slight decrease in the protective effect of quercetin with increasing concentration was due to its toxicity, the effect of quercetin was retested in the lower concentration range (0.1–10 μM) (Figure 3b). Quercetin showed significant and concentration-dependent protective effects, without cytotoxicity. IC_50_ values of quercetin and isoquercitrin were calculated to be 7.0 ± 0.2 and 56.1 ± 2.8 μM, respectively. Since apoptosis was demonstrated to be the dominant cell death mechanism in the Glu-HT22 assay system [17], we next examined whether isolated compounds modulate Glu-induced apoptosis. After HT22 cells were incubated with drugs for 8 h, cell death was measured using PI/Annexin V staining and flow cytometry (Figure 3c,d). Depending on the fluorescence intensities of PI and Annexin V in the dot plot of normal cells, quadrants were assigned to distinguish the cell death modalities including apoptosis and necrosis, that is, intact cells (PI^−^/Annexin V^−^, Q1), early apoptosis (PI^−^/Annexin V^+^, Q2), late stage cell death of both necrosis and apoptosis (PI^+^/Annexin V^+^, Q3), and necrosis (PI^+^/Annexin V^−^, Q4). The apoptotic cell population (%) was expressed as the sum of Q2 and Q3. A significant increase of apoptotic cells was found in Glu control cells. Quercetin and isoquercitrin significantly decreased apoptotic cells in a concentration-dependent manner, suggesting that they inhibited Glu-induced apoptosis in HT22 cells. Consistent with the cell viability assay, the other compounds did not show inhibitory effects on apoptosis at 100 μM.

### 3.4. Isoquercitrin and Quercetin Attenuate Glu-Induced Intracellular/Mitochondrial ROS Generation and Ca^2+^ Dysregulation

Oxidative stress and Ca^2+^ dysregulation are involved in excessive extracellular Glu-induced neuronal cell death in the nervous system [6,7]. The accumulation of reactive radical species and increase in Ca^2+^ levels can disrupt cellular homeostasis for adaptation and survival. The signaling of ROS and Ca^2+^ are complexly entangled by mutual crosstalk and influence each other [35]. We demonstrated that intracellular levels of ROS and Ca^2+^ are almost simultaneously elevated after Glu challenge in HT22 cells [17]. Thus, we examined the effect of isoquercitrin and quercetin on ROS generation and Ca^2+^ dysregulation caused by Glu.

**Figure 4:**
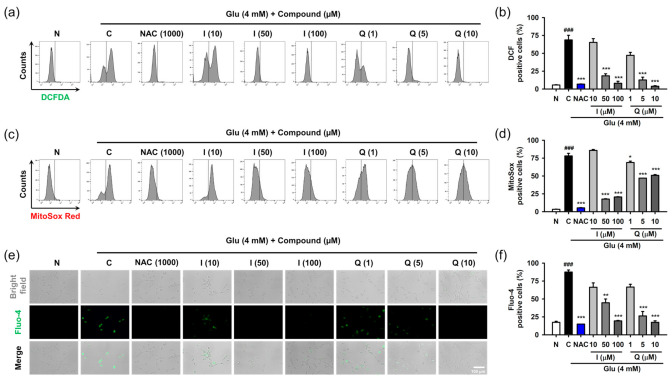
Effect of isoquercitrin and quercetin on the Glu-induced ROS generation and Ca^2+^ dysregulation in HT22 cells. (**a**,**b**) Cellular ROS generation measured using CM-H_2_DCF-DA staining and flow cytometric analysis; (**c**,**d**) Mitochondrial ROS generation measured using MitoSox Red staining and flow cytometric analysis; (**e**,**f**) Cellular Ca^2+^ levels measured using Furo-4 staining and fluorescence microscopic analysis. After HT22 cells were incubated with compound or Glu for 8 h, cells were stained and analyzed; Student’s *t*-test (N vs. C, ^###^ *p* < 0.001); One-way ANOVA (C vs. Glu + drugs, * *p* < 0.05, ** *p* < 0.01, *** *p* < 0.001); CGA, chlorogenic acid; CM-H_2_DCF-DA, 2′,7′-dichlorodihydrofluorescein diacetate chloromethyl derivative; H, hyperoside; I, isoquercitrin; Q, quercetin; R, rutin; ROS, reactive oxygen species; Glu, glutamate; N, non-treated normal cell; C, control cells treated with Glu only; PI, propidium iodide.

After HT22 cells were treated with 4 mM Glu and/or compounds for 8 h, ROS and Ca^2+^ levels were determined using specific fluorescence probes such as CM-H_2_DCF-DA for cellular ROS, MitoSox Red for mitochondrial ROS, and Furo-4 for cellular Ca^2+^ (Figure 4). NAC was used as the positive control. We previously confirmed that 1 mM NAC was non-cytotoxic and completely inhibited Glu-induced HT22 cell death [17]. Glu significantly increased cellular and mitochondrial ROS levels as well as Ca^2+^ levels, and this effect was significantly reversed by NAC. These data suggest that oxidative stress and Ca^2+^ dysregulation are causative of Glu toxicity. As expected, isoquercitrin and quercetin significantly restored Glu-induced ROS generation and Ca^2+^ increase in a concentration-dependent manner, indicating that the antioxidant effect mediates the protective effects of isoquercitrin and quercetin on Glu-induced oxidative cell death. Of note, Glu-induced mitochondrial ROS generation and intracellular Ca^2+^ increase may imply functional defects in mitochondria; thus, it was necessary to examine the effect of isoquercitrin and quercetin on the restoration of Glu-induced mitochondrial dysfunction.

### 3.5. Isoquercitrin and Quercetin Restore Glu-Induced Mitochondrial Dysfunction

Mitochondria are critical cellular organelles that not only sustain survival but also regulate intrinsic cell death mechanisms. As a result of the high demand for energy and oxygen, mitochondrial bioenergetic function is even more important to the high rate of metabolism and physiological function of the brain [36,37]. Oxidative stress and mitochondrial dysfunction are responsible for neuronal cell death associated with aging, as well as many neurological disorders such as stroke, traumatic brain injury, and neurodegenerative diseases [38,39]. Mitochondrial dysfunction has been identified in Glu-induced oxidative cell death in HT22 cells [6,17]. Therefore, we investigated whether isoquercitrin and quercetin were able to restore Glu-induced mitochondrial dysfunction.

**Figure 5:**
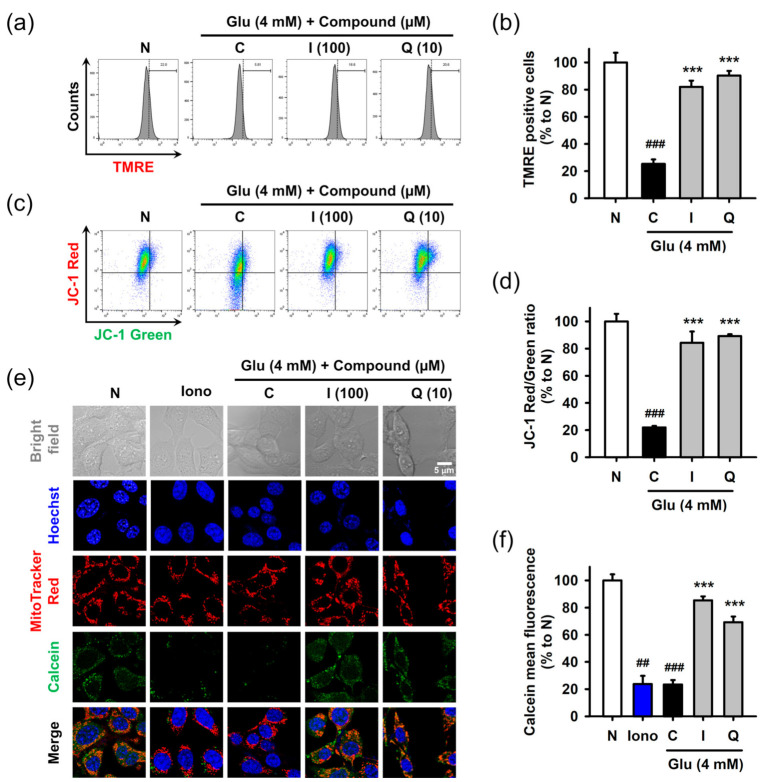
Effect of isoquercitrin and quercetin on the Glu-induced mitochondrial dysfunction in HT22 cells. (**a**,**b**) Mitochondrial membrane potential measured using TMRE staining and flow cytometric analysis; (**c**,**d**) Mitochondrial membrane potential measured using JC-1 staining and flow cytometric analysis; (**e**,**f**) Mitochondrial membrane permeabilization measured using Hoechst/MitoTracker Red/calcein AM/Co^2+^ staining and confocal microscopic analysis; After HT22 cells were incubated with compound or Glu for 8 h, cells were stained and analyzed; Student’s *t*-test (N vs. C, ^##^ *p* < 0.01, ^###^ *p* < 0.001); One-way ANOVA (C vs. Glu + drugs, *** *p* < 0.001); I, isoquercitrin; Q, quercetin; Glu, glutamate; N, non-treated normal cell; C, control cells treated with Glu only; Iono, ionomycin; JC-1, 5,5′,6,6′-tetrachloro-1,1′3,3′-tetraethylbenzamidazol-carboncyanine; TMRE, tetramethyl rhodamine ethyl ester.

Mitochondrial dysfunction was assessed in terms of the mitochondrial membrane potential and permeability transition using fluorometric assays. After treatment with 4 mM Glu and/or compounds for 8 h, HT22 cells were used for each assay. Mitochondrial membrane potential was measured using potential sensitive dyes, including TMRE (Figure 5a,b) or JC-1 (Figure 5c,d), followed by flow cytometric analysis. Glu significantly decreased signals of both TMRE and JC-1 red (aggregate form found in polarized mitochondria) and increased the signal of JC-1 green (monomer form found in depolarized mitochondria). These results suggested that Glu caused mitochondrial depolarization. Isoquercitrin and quercetin significantly reversed Glu-induced mitochondrial depolarization. Mitochondrial membrane permeability transition was measured using Hoechst/MitoTracker Red/calcein AM/Co^2+^ staining followed by confocal microscopic analysis (Figure 5e,f). Ionomycin, a trigger for mitochondrial pore opening, was used as a positive control. Since cytosolic calcein was quenched by cytosolic Co^2+^, the green punctate dot of calcein, which exactly overlapped with the red dot of MitoTracker Red, represents normal intact mitochondria. Ionomycin and Glu decreased mitochondrial calcein fluorescence, suggesting that they induced mitochondrial membrane permeabilization, leading to the influx of Co^2+^ into mitochondria and quenching of mitochondrial calcein. Co-treatment of isoquercitrin or quercetin with Glu significantly increased calcein signals, suggesting that these two prevented Glu-induced mitochondrial membrane permeabilization. Collectively, isoquercitrin or quercetin alleviated Glu-induced mitochondrial dysfunction by sustaining mitochondrial polarity and membrane integrity.

### 3.6. Isoquercitrin and Quercetin Inhibit Glu-Induced Nuclear Translocation of AIF

Mitochondrial membrane permeabilization, which refers to irreversible damage to membrane integrity, is often regarded as an initiation of an intrinsic cell death mechanism called mitochondrial apoptosis [40,41]. Permeabilization results in the release of mitochondrial apotogenic proteins, triggering the cell death cascade via caspase- or apoptosis-inducing factor (AIF)-dependent mechanisms. In particular, AIF-dependent cell death is known to be involved in cerebrovascular and neurodegenerative diseases [42,43]. Nuclear translocation of AIF is a major contributor to Glu-induced oxidative cell death in HT22 cells [6,17,44]. This was confirmed by an experiment using pharmacological inhibitors (Figure S12). The AIF translocation inhibitor necrostatin-1 reversed Glu-induced loss of cell viability, but the caspase inhibitor z-VAD-fmk failed to reverse. Therefore, we examined whether isoquercitrin or quercetin prevented Glu-induced nuclear translocation of AIF in HT22 cells.

**Figure 6:**
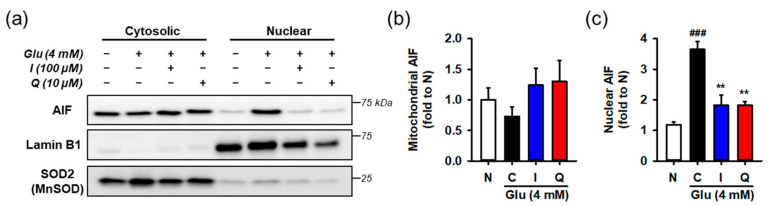
Effect of isoquercitrin and quercetin on the Glu-induced nuclear translocation of AIF. (**a**) Representative blot images; (**b**,**c**) Quantitative data of mitochondrial and nuclear levels of AIF; After incubation with compounds or Glu for 12 h, HT22 cells were fractionated into cytosolic and nuclear fraction. Lysates of each fraction were analyzed using Western blotting; Student’s *t*-test (N vs. C, ^###^ *p* < 0.001); One-way ANOVA (C vs. Glu + drugs, ** *p* < 0.01); AIF, apoptosis-inducing factor; I, isoquercitrin; Q, quercetin; Glu, glutamate; N, non-treated normal cell; C, control cells treated with Glu only.

After HT22 cells were incubated with 4 mM Glu and/or the compounds for 12 h, cytosolic and nuclear fractions was prepared from harvested cells. AIF nuclear translocation was assessed by measuring the levels of proteins in each fraction using Western blotting (Figure 6). To demonstrate the performance of fractionation or interpret the blot data, lamin-b and SOD2 were used as nuclear and mitochondrial markers or loading controls, respectively. Since the contamination of mitochondria in the nucleus fraction may exaggerate the increase of AIF nuclear translocation, SOD2 instead of tubulin was used for the cytosolic marker. Glu significantly increased nuclear levels of AIF, whereas isoquercitrin or quercetin reversed Glu-induced nuclear translocation of AIF. These data imply that maintenance of the functional and structural integrity of mitochondria underlies the pro-survival effect of isoquercitrin or quercetin on Glu-induced HT22 cell death.

### 3.7. Isoquercitrin and Quercetin Activate Nrf2/HO-1 Signaling Pathway

Oxidative stress imposes an excessive burden on intrinsic antioxidant defense mechanisms, resulting in an imbalance in redox homeostasis. Nrf2 is a transcriptional master regulator of neuronal resistance to oxidative stress and Glu-induced excitotoxicity in the nervous system [45]. Nrf2 accumulation to facilitate binding to ARE requires decomposition of the Keap1/Nrf2 complex. Under conditions of oxidative stress, ROS modify the structure of Keap1, which is a thiol-rich protein with many cysteine residues vulnerable to attack by electrophiles, resulting in an increase in free Nrf2 levels in the cytoplasm. Nrf2 stabilization is involved in the recovery of cellular antioxidant defenses via the upregulation of antioxidant enzymes and proteins, increase in redox transport, such as system $x_{c}^{-}$, and induction of stress response proteins, such as heme oxygenase 1 (HO-1) [46]. HO-1, an Nrf2-dependent gene that is highly upregulated following lethal stimuli, such as oxidative stress, is known to be cytoprotective and reduces stress by generating antioxidant and antiapoptotic molecules, such as CO and biliverdin [10]. Under autophagy deficiency, excess p62 directly interacts with the Nrf2-binding site on Keap1 and competes with Nrf2, resulting in Nrf2 stabilization, nuclear translocation, and transcription [47]. Since autophagy is involved in the degradation and turnover of free Keap1, the autophagy pathway is critical in sustaining the integrity of the Keap1/Nrf2 axis [48]. Some dietary polyphenols and natural products have been reported to modulate the Nrf2 pathway [15,16]. Glu facilitated ROS generation in HT22 cells, as shown in Figure 4, and it is known to induce autophagy activation [49]. Therefore, we examined the effect of the isolated compounds on the pathways of Keap1/Nrf2/HO-1 and autophagy.

**Figure 7:**
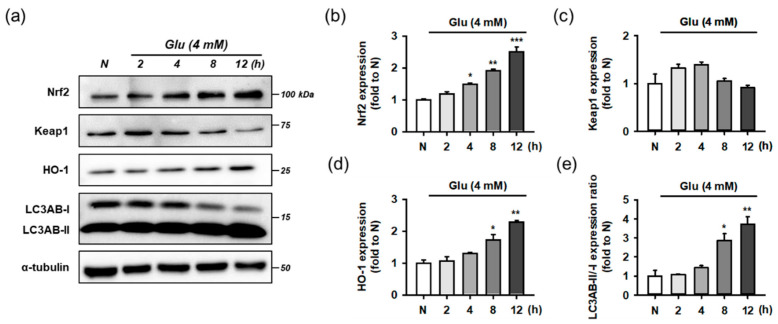
Effect of Glu on Nrf2/HO-1 and autophagy pathways. (**a**) Representative blot images; (**b**–**e**) Quantitative data of protein levels of Nrf2, Keap1, HO-1, and ratio of LC3AB-II/-I; After HT22 cells were incubated with compound or Glu for 0–12 h, cell lysates were analyzed using Western blotting; One-way ANOVA (N vs. Glu, * *p* < 0.05, ** *p* < 0.01, *** *p* < 0.001); Glu, glutamate; N, non-treated normal cell; HO-1, heme oxygenase 1; Keap1, Kelch-like ECH associating protein; Nrf2, nuclear factor erythroid-2-related factor 2.

Before testing the effect of the compounds, we examined whether Glu modulates the Nrf2 and autophagy pathways as an adaptive response to oxidative stress. For time-course experiments, HT22 cells were treated with 4 mM Glu for 0–12 h. Protein levels of Keap1, Nrf2, HO-1, LC3AB, and α-tubulin were measured using Western blotting (Figure 7). The activation of autophagy was assessed by the conversion of LC3AB-I to LC3AB-II (LC3AB-II/-I ratio). Glu increased the levels of Nrf2, HO-1, and ratio of LC3AB-II/-I in a time-dependent manner, but the Keap1 level was slightly increased at early times from 2 to 4 h and reduced to normal levels after 8 h. Considering the significant autophagy activation after 8 h of Glu treatment, autophagy may partly contribute to the maintenance of even levels of cytosolic Keap1 as the clearance and recycling machinery. As a result of rapid proteasomal degradation, Nrf2 has a relatively short half-life of 20 min compared to Keap1 at 12 h [11]. The trend of Nrf2 and HO-1 expression following Glu challenge was exactly the same, implying that the excess cytoplasmic Nrf2 was readily translocated to the nucleus and increased HO-1 expression. These data suggest that Glu significantly activates Nrf2 signaling and autophagy after 8 h.

**Figure 8:**
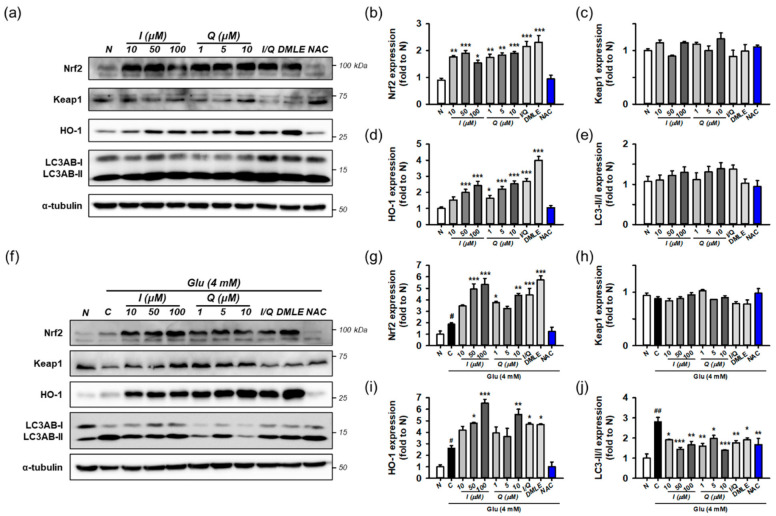
Effect of isoquercitrin and quercetin on the Nrf2/HO-1 and autophagy pathways. (**a**) Representative blot images showing the effect of compounds without Glu treatment; (**b**–**e**) Quantitative data of protein levels of Nrf2, Keap1, HO-1, and ratio of LC3AB-II/-I in blot (**a**); (**f**) Representative blot images showing the effect of compounds with Glu treatment; (**g**–**j**) Quantitative data of protein levels of Nrf2, Keap1, HO-1, and ratio of LC3AB-II/-I in blot (**f**); After HT22 cells were incubated with compound or Glu for 8 h, cell lysates were analyzed using Western blotting; Student’s *t*-test (N vs. C, ^#^ *p* < 0.05, ^###^ *p* < 0.001); One-way ANOVA (C vs. Glu + drugs, * *p* < 0.05, ** *p* < 0.01, *** *p* < 0.001); Glu, glutamate; N, non-treated normal cell; C, control cells treated with Glu only; I, isoquercitrin; Q, quercetin; I/Q, combination of 50 μM of I and 7.0 μM of Q; DMLE, 100 μg/mL of ethyl acetate-soluble extracts of *Dendropanax morbifera* leaves; NAC, 1 mM of *N*-acetyl-L-cysteine.

Next, the effects of isoquercitrin or quercetin on Keap1/Nrf2/HO-1 and autophagy were examined in the absence of Glu. HT22 cells were incubated for 8 h with isoquercitrin (10–100 μM), quercetin (1–10 μM), or I/Q (combination of isoquercitrin 50 μM and quercetin 7.0 μM, each of which corresponds to approximate IC_50_ values in the MTT assay). Protein levels were determined using Western blotting (Figure 8a–e). The effects were compared with those of DMLE (100 μg/mL) and NAC (1 mM). Isoquercitrin or quercetin significantly increased Nrf2 and HO-1 levels by approximately 2-fold in a concentration-dependent manner. This was also consistently found with I/Q and DMLE but not in NAC. These findings suggest that the activation of the Nrf2/HO-1 pathway may be associated with the protective effects of isoquercitrin or quercetin, which can be differentiated from those of NAC. However, none of the drugs changed Keap1 levels or the LC3AB-II/-I ratio. Although it may not be sufficient to explain the effect on autophagy with only the LC3AB-II/-I ratio, these data suggest that all compounds have no effect on the autophagy pathway and that excess Keap1 seems to be recycled by the basal autophagic flux in HT22 cells.

Finally, the effects of isoquercitrin or quercetin on Keap1/Nrf2/HO-1 and autophagy were examined in the presence of 4 mM Glu under the same experimental conditions (Figure 8f–j). Glu increased the levels of Nrf2, HO-1, and the ratio of LC3AB-II/-I, which is consistent with Figure 7. NAC restored the effects of Glu to almost normal levels. Interestingly, co-treatment with isoquercitrin, quercetin, I/Q, and DMLE with Glu significantly increased Nrf2 and HO-1 levels compared to Glu alone and decreased the LC3AB-II/-I ratio to a level lower than Glu alone. A significant change in Keap1 was not observed in any of the treatment groups. Taken together, these findings imply that Nrf2 signaling and autophagy mediate Glu-induced oxidative cell death. The activation of Nrf2 signaling and modulation of autophagy may be protective mechanisms of isoquercitrin or quercetin.

### 3.8. Molecular Docking Simulation of Isoquercitrin and Quercetin to Keap1-BTB Domain

**Figure 9:**
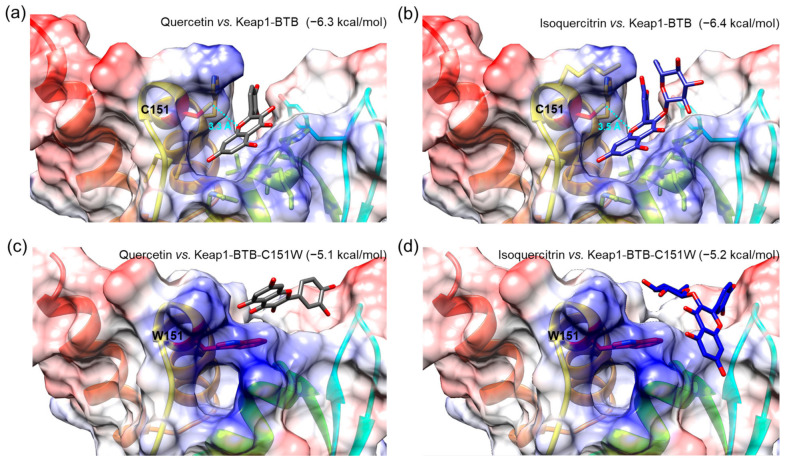
Molecular docking of Q and I with BTB domain of Keap1 (Keap1-BTB, (**a**,**b**)) or mutant BTB domain at C151W of Keap1 (Keap1-BTB-C151W, (**c**,**d**)). A compound and protein pair was presented in each image, and the value in parentheses indicates binding free energy as a unit of kcal/mol. A sky-blue dotted line indicates the distances between oxygen at the 1-position in both compounds and cysteine residue (C151) of Keap1-BTB, and the value represents the distance as a unit of Angstrom (Å). Tryptophan residue (W151) was indicated in the mutant Keap1-BTB-C151W. Chemical bonds of Q and I are depicted as gray and blue sticks, respectively. The residues with negatively and positively charged residues are colored red and blue, respectively.

To investigate the interactions of isolated compounds with the active site in the BTB domain of Keap1 (Keap1-BTB), we performed a molecular docking simulation. The binding free energies of isoquercitrin or quercetin for Keap1-BTB binding were −6.4 and −6.3 kcal/mol, respectively (Figure 9), which were lower (i.e., more stable) than that of other compounds (hyperoside, −5.7 kcal/mol; rutin, −6.1 kcal/mol; chlorogenic acid, −6.1 kcal/mol; Figure S14). In our docking model, quercetin was located in the binding pocket of Keap1-BTB with the same orientation as the previously reported docking model (Figure 9a) [50]. The hydroxyl at the C-7 position of quercetin was located inside the binding pocket, and the hydroxyl groups at C-3′ and C-4′ were facing outward. The relative orientation of isoquercitrin was almost identical to that of quercetin; therefore, glucose attached at C-3 was located on the opposite side of the binding pocket (Figure 9b). Oxygen at the 1-position (O-1) of both quercetin and isoquercitrin was the closest atom to cysteine residue (C151) in Keap1, and the distances between the two were 3.3 Å for quercetin and 3.5 Å for isoquercitrin. Furthermore, our docking models indicated that the hydroxyl groups at C-7 of quercetin and isoquercitrin are closely associated with the positively charged residues in the binding pocket of Keap1-BTB (blue regions in Figure 9a,b), thus stabilizing the binding of quercetin and isoquercitrin to Keap1. The importance of C151 for ligand binding was also revealed by the docking of quercetin and isoquercitrin to the mutant BTB domain at C151W of Keap1 (Keap1-BTB-C151W; Figure 9c,d). Due to steric hindrance by the bulky side chain of the tryptophan residue (W151), the structure of the binding pocket was modified to restrict the access of ligands. The relative orientations of the compounds were completely different from those of Keap1-BTB, and compounds could not fit into the binding pocket. Isoquercitrin and quercetin docked in mutant Keap1-BTB showed completely different conformation with higher binding free energies (−5.2 kcal/mol for isoquercitrin and −5.1 kcal/mol for quercetin), suggesting weak and unstable binding. Therefore, the effect of isoquercitrin and quercetin on Nrf2 activation may be mediated by their interaction with Keap1.

### 3.9. Identification of Active Principles in DMLE Using HPLC Assay and Combinatory Treatment

Due to the diversity of phytochemicals in the plant extracts, it can be difficult to assume that a single active ingredient represents the entire activity of the extract. The beneficial bioactivity of the extract can be manifested by the various interactions of the component chemicals or the capability of multi-targeting the relevant biological molecules. In particular, since the therapeutic effect is closely related to the dose, it is important to determine the content of constituents in the extract.

**Figure 10:**
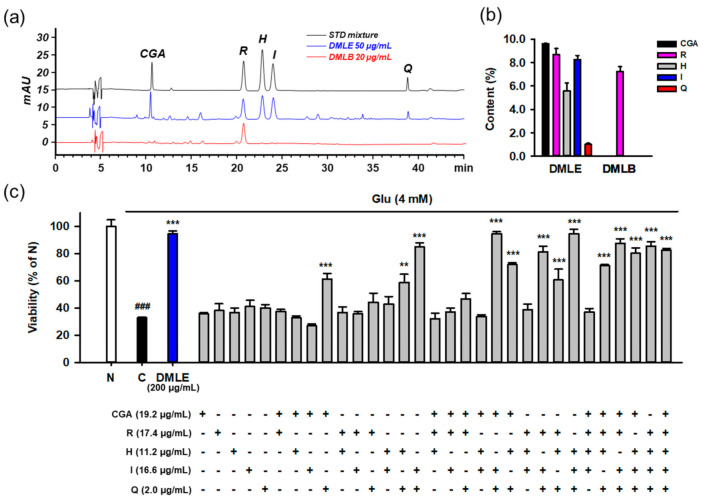
Determination of isolated compounds in the extracts of *Dendropanax morbifera* leaves and identification of active ingredients representing the protective effects of extracts using the combinatory treatment. (**a**) HPLC-UV chromatograms of standard mixture (5 μg/mL for CGA, R, H, and I; 0.5 μg/mL for Q) and solvent extracts of DML (50 μg/mL for DMLE; 20 μg/mL for DMLB); (**b**) Contents (%, *w*/*w*) of isolated compounds in solvent extracts of DML. Bar represents mean ± standard deviation; (**c**) Protective effects of isolated compounds on the Glu-induced HT22 cell death using the combinatory treatment strategy. HT22 cells were treated with Glu and/or diverse combinations of compounds for 24 h. Indicated concentrations of compounds were equivalent to their contents in 200 μg/mL of DMLE. Cell viability was measured using MTT assay; Student’s *t*-test (N vs. C, ^###^ *p* < 0.001); One-way ANOVA (C vs. Glu + drugs, ** *p* < 0.01, *** *p* < 0.001); DML, *Dendropanax morbifera* leaves; DMLE, ethyl acetate-soluble extracts of DML; DMLB, *n*-butanol soluble extracts of DML; CGA, chlorogenic acid; H, hyperoside; I, isoquercitrin; Q, quercetin; R, rutin; Glu, glutamate; N, non-treated normal cell; C, control cells treated with Glu only.

To identify the active compounds that represent the protective activity of DMLE as a whole, we determined the contents of the isolated compounds using HPLC-UV assay. The developed assay showed acceptable method performance characteristics in terms of specificity, linearity, LOD, LOQ, precision, and accuracy. Typical chromatograms of the standards and two samples, including DMLE and DMLB, are shown in Figure 10a. All the compounds were completely resolved. The retention time and UV spectra of each compound were identical between the standard and sample, implying that this method was specific without interference from matrix components. The calibration curves showed excellent linearity (R^2^ ≥ 0.9995) within the specified range (Table S2). The LOD and LOQ ranged from 0.01 to 0.13 μg/mL. In the intra- and inter-day assays, the relative standard deviation (RSD) and recovery of analytes, which were calculated from standard spiked replicates, were within 0.2–8.3% and 97.0–133.2%, respectively, suggesting that this assay showed excellent precision and accuracy. The contents (% *w*/*w*, mean ± standard deviation) of the five isolated compounds were determined in DMLE and DMLB (Figure 10b). Chlorogenic acid (9.6 ± 0.2%) was the major compound in DMLE, followed by rutin (8.7 ± 0.5%), isoquercitrin (8.3 ± 0.3%), hyperoside (5.6 ± 0.7), and quercetin (1.0 ± 0.1%). Only rutin (7.3 ± 0.4%) was present in DMLB. Due to a polar disaccharide residue, rutin can also be partitioned to *n*-butanol fraction, which is the most polar solvent among four solvents used in our fractionation.

To identify the active principles representing the protective activity of DMLE, we prepared an artificial DMLE consisting of part or all of the five compounds and further examined the protective effects on Glu-induced HT22 cell death. Based on the contents determined above, the concentration of each compound was adjusted to a concentration corresponding to 200 μg/mL of DMLE, i.e., chlorogenic acid (19.2 μg/mL), rutin (17.4 μg/mL), hyperoside (11.2 μg/mL), isoquercitrin (16.6 μg/mL), and quercetin (2.0 μg/mL). A total of 31 combinations were possible, including one to all five. HT22 cells were treated with each combination for 24 h, and cell viability was determined using the MTT assay (Figure 10c). The combinatorial treatment groups containing both isoquercitrin and quercetin mostly showed significant protective effects. Since significant protective effects were also observed in some combinations containing chlorogenic acid, isoquercitrin, and quercetin, we examined the effects of drug–drug interactions between chlorogenic acid and isoquercitrin or quercetin. Cells were treated with isoquercitrin (10–100 μM) or quercetin (1–10 μM) alone or in combination with chlorogenic acid (25–100 μM) in the presence of 4 mM Glu. After incubation for 24 h, the cell viability was determined. No significant improvement in protection was found between isoquercitrin or quercetin alone and in combination with chlorogenic acid (Figure S15). Notable toxicities were found with the combination of quercetin (5–10 μM) and chlorogenic acid (100 μM). These data suggest that isoquercitrin and quercetin are active ingredients that represent the entire activities of DMLE.

### 3.10. Combination of Isoquercitrin and Quercetin Synergistically Inhibited Glu-Induced Cell Death

**Figure 11:**
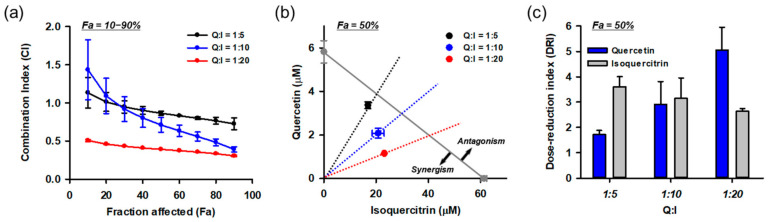
Synergistic effects between isoquercitrin and quercetin resulting in the protection of HT22 cells against Glu-induced toxicity. (**a**) Combination index (CI) analysis at the diverse effect levels (Fa, fraction affected; inhibitory activity). CI = 1, additivity; CI > 1, antagonism; CI < 1, synergy; (**b**) Isobologram analysis at 50% Fa. Gray diagonal line indicates additive isobole. Symbols show concentration of drugs at 50% Fa. The area of synergism and antagonism was indicated by arrow; (**c**) Dose-reduction index (DRI) at Fa = 0.5; Symbols and bars represent mean ± standard deviation; I, isoquercitrin; Q, quercetin.

Based on the HPLC and combinatory treatment assays, we found that isoquercitrin and quercetin are responsible for the protective effects of DMLE. Since they coexist in DMLE at a molar ratio of quercetin:isoquercitrin = 1:5.4, it was assumed that the effect of DMLE may be influenced by the drug–drug interactions between the two. Therefore, we quantitatively evaluated the combination effect using the Chou–Talalay method. Since interactions between the drugs can vary with their relative concentrations, we tested the effects at multiple molar concentration ratios (quercetin:isoquercitrin = 1:5, 1:10, 1:20) covering their naturally occurring ratio in DMLE. Each drug was treated at the same time as the control. Drug–drug interaction data analyzed using CompuSyn software are shown in Figure 11. The combination index (CI) was calculated at various effect levels (Fa, inhibitory activity; Figure 11a). The CI values changed depending on the Fa and quercetin/isoquercitrin ratio, and they showed a decreasing trend as Fa increased. Synergism (CI < 1) was found in 50% Fa in all combinations and increased with Fa and the ratio of isoquercitrin. Since the efficacy of a drug is frequently assessed at its IC_50_, an isobologram was constructed at 50% Fa (Figure 11b). The isoboles of all combinations were below the additive isobole (gray diagonal line in Figure 11b) and decreased further as the relative concentration of isoquercitrin increased. The dose-reduction index (DRI), which indicates a fold reduction in drug concentration in combination with that of the drug alone at the same effect levels, was also calculated at 50% Fa (Figure 11c). As the relative concentration of isoquercitrin increased in the combination, the DRI values of quercetin increased from 1.7 to 5.0, but those of isoquercitrin decreased from 3.6 to 2.6. The overall dose reduction was maximum at quercetin:isoquercitrin = 1:20. These data suggest that the synergistic effect of isoquercitrin and quercetin contributes to the neuroprotective effect of DMLE.

## 4. Discussion

Following our previous studies on the neuroprotective effects of DMLE at the extract level, we investigated the active ingredients and mechanism of protection of DMLE. To this end, we systematically organized multidisciplinary experiments and performed the following in a step-by-step manner: isolation and structural identification of compounds, mechanism study using pharmacological and biochemical tools, chemical analysis of their contents, and quantitative drug–drug interaction studies. Five antioxidant compounds were identified, including chlorogenic acid, quercetin, and its glycosides (isoquercitrin, hyperoside, and rutin). Hyperoside and isoquercitrin were identified in DM for the first time. Isoquercitrin and quercetin were demonstrated to be the active ingredients representing the entire activity of DMLE via Nrf2/HO-1 activation. Importantly, these two synergistically protected HT22 cells against Glu-induced oxidative cell death. Since more than half of new medicines approved during recent decades were derived from natural products or their derivatives, natural products as the infinite sources of new drugs are still inspiring the development of innovative pharmacophores [51]. Owing to many issues, such as patents and safety, novelty and potency have been the focus of interest for new drug development from natural sources, regardless of their ethnopharmacological relevance and abundance. Since dietary polyphenols and other major phytochemicals that have been frequently exposed to humans are ubiquitous and abundant in natural products, they may have a great impact on human health and evolution as well as coexistence among the species for a long time. Our findings are not only related to the traditional efficacy of DML for alleviating pain in the brain but also show that the active ingredients can cooperatively contribute to its efficacy.

The protective effects against Glu-induced oxidative HT22 cell death were only found in quercetin aglycone and its 3-O glucoside (isoquercitrin). Despite the structural similarities, except for chlorogenic acid, 3-O rutinoside (rutin) and 3-O galactoside (hyperoside) failed to reverse Glu toxicity. Quercetin showed mild toxicity at higher concentrations, which is consistent with the literature [52], but the others showed no toxicity within the test ranges. Since quercetin aglycone has structural features responsible for its excellent radical scavenging capability, all of them showed potent and comparable antioxidant effects in cell-free antioxidant assays. In the docking simulation with Keap1-BTB, they also showed a similar orientation and low free energies, and oxygen at the 1-position in all aglycone moieties is the critical element in the interaction with C151 of Keap1-BTB. These results imply that all quercetin derivatives may exert similar potency if they can freely enter HT22 cells. Differences in cell membrane penetration or cellular uptake may be the possible factors explaining the differential protection by quercetin derivatives on HT22 cells. The cellular absorption of quercetin and its glycosides can vary depending on their structural features and cell types [52,53,54]. Cellular absorption of quercetin was also found in the primary cerebellar granule neurons; however, data on other glycosides are lacking in the literature [55]. In the intestinal absorption model using human colon adenocarcinoma Caco-2 cells, quercetin is known to exhibit a higher absorption than quercetin glycosides (isoquercitrin, hyperoside, and rutin) [56,57,58]. Both Caco-2 cells treated with isoquercitrin alone accumulated both quercetin and isoquercitrin within an hour, suggesting that isoquercitrin is readily metabolized to quercetin by cellular glucosidases [57]. Dietary flavonoids undergo diverse metabolism in the intestinal tract by enzymes such as lactase-phlorizin hydrolase and cytosolic glucosidases as well as microbiota [52,53,59]. This metabolic transformation may influence the absorption and bioavailability of individual quercetin derivatives in a more intensive and complicated manner, in plant extracts composed of diverse flavonoids [52,57]. Rats fed with isoquercitrin showed higher levels of metabolites in plasma and other tissues, including the brain, than those fed with quercetin, suggesting that the bioavailability of isoquercitrin is higher than that of quercetin, and orally administered isoquercitrin can be a source of quercetin in the body [60]. DMLE contained approximately 8-fold higher levels of isoquercitrin than quercetin. Isoquercitrin or quercetin alone has been reported to exhibit protective effects in an ischemic stroke model in vivo [61,62]. These findings suggest a positive prospect for the neuroprotective effects of DMLE on the neuropathological animal models associated with Glu toxicity in our future study.

Nrf2/HO-1 activation and autophagy modulation were found to be protective mechanisms of DMLE. This was comprehensively confirmed by single compound levels in the presence or absence of Glu cotreatment. Numerous studies have demonstrated that isoquercitrin and quercetin are responsible for the activation of the Nrf2 signaling pathway [52,63]. Since autophagy is involved in the turnover of free Keap1, it was monitored in our study in terms of LC3 conversion. Autophagic cell death has been reported to cause cell death in HT22 cells treated with 5 mM Glu [49]. Consistently, we found that Glu induced persistent and increased LC3 conversion. DMLE and its active ingredients significantly reversed Glu-induced LC3 conversion. The change in LC3 conversion is not sufficient to conclude either autophagy activation or inhibition due to its dependence on autophagic flux [64]. Autophagy plays multiple roles in cellular survival and death [65]. Autophagy can be adaptive for survival in response to sublethal or transient stimuli, but it can be decisive to cell death following intensive and persistent stress. Moreover, these contradictory roles regulating life and death can change as a function of the intensity and duration of stress. Active compounds showed antioxidant effects, such as rapid radical scavenging and Nrf2/HO-1 activation as described in the hypothetical model of protective mechanisms (Figure S16), implying that the strength of Glu-induced oxidative stress may be minimized by reducing the ROS levels via direct scavenging and/or activation of intrinsic antioxidant defenses. Considering these points, the exact role of autophagy needs to be further evaluated in the protection of active ingredients and DMLE.

Quantitative evaluation of the synergism between isoquercitrin and quercetin was a major finding in our study. Without reliable quantitation of isolated compounds in DMLE and their combined treatment equivalent to their contents, we might have missed the drug–drug interactions between the two. Our findings suggest the importance of chemical analysis to understand the beneficial activities of plant extracts, the so-called nature’s drug prescription. The data of chemical analysis can be even more valuable for discovering unexposed phenomena if they are applied and evaluated by biological assessment. Moreover, the validated HPLC assay can be utilized for the standardization of DMLE using isoquercitrin and quercetin as markers. Synergism was found at all concentration ratios and increased with the concentration ratio of isoquercitrin in the combination. Considering that the inherent ratio of the two in DMLE is within the test ranges, isoquercitrin and quercetin represent the protective effect of DMLE as a whole via effective synergism. Unlike isoquercitrin, quercetin showed double-edged cellular responses depending on the concentration, that is, potent protection at low, and mild toxicity at high concentrations, indicating a narrow therapeutic window. The metabolic transformation machinery of flavonoid converted isoquercitrin to quercetin, contributing to the maintenance of appropriate concentrations of quercetin to only result in the therapeutic effect without toxicity. Metabolic transformation also contributes to sustaining their concentrations for a long and delayed time. These can be reasonable and comprehensive speculations explaining the synergism between the two, as well as the differential synergistic effects depending on the concentration ratios. Rutin and hyperoside were not effective but were abundant in DMLE. Both can also be sources of quercetin and isoquercitrin through metabolic transformation in the digestive organs. The absorption of flavonoids can differ according to the cell type. Therefore, further studies are needed using systemic approaches to evaluate the absorption, metabolism, pharmacokinetics, drug interactions, and therapeutic efficacies in diverse cell types or animal models. This may contribute to expanding our insight into a better understanding of the therapeutic mechanisms of DMLE or quercetin derivatives.

## 5. Conclusions

In this study, we demonstrated that isoquercitrin and quercetin are active compounds that represent the neuroprotective effects of DMLE on Glu-induced oxidative HT22 cell death. Active compounds activate the Nrf2/HO-1 pathway, which is implicated in the protective mechanism of action. Importantly, the synergism between the two was quantitatively confirmed by drug combination analysis. We conclude that *Dendropanax morbifera* leaves can be a viable therapeutic option for neurological disorders induced by Glu toxicity.

## Data Availability

Not applicable.

## Supplementary Materials

The following are available online at https://www.mdpi.com/article/10.3390/antiox10040554/s1, Figure S1: 1H-NMR spectrum of compound **1** (quercetin, 500 MHz, DMSO-d6), Figure S2: 13C-NMR spectrum of compound **1** (quercetin, 125 MHz, DMSO-d6), Figure S3: 1H-NMR spectrum of compound **2** (isoquercitrin, 500 MHz, DMSO-d6), Figure S4: 13C-NMR spectrum of compound **2** (isoquercitrin, 125 MHz, DMSO-d6), Figure S5: 1H-NMR spectrum of compound **3** (hyperoside, 500 MHz, DMSO-d6), Figure S6: 13C-NMR spectrum of compound **3** (hyperoside, 125 MHz, DMSO-d6), Figure S7: 1H-NMR spectrum of compound **4** (rutin, 500 MHz, DMSO-d6), Figure S8: 13C-NMR spectrum of compound **4** (rutin, 125 MHz, DMSO-d6), Figure S9: 1H-NMR spectrum of compound **5** (chlorogenic acid, 400 MHz, DMSO-d6), Figure S10: 13C-NMR spectrum of compound **5** (chlorogenic acid, 175 MHz, DMSO-d6), Figure S11: LC-ESI-MS total ion chromatogram of DMLE and mass spectra of isolated compounds, Figure S12: Effect of z-VAD-fmk and Necrostatin-1 on the Glu-induced HT22 cell death, Figure S13: Effect of isolated compounds on the Nrf2/HO-1 and autophagy pathways, Figure S14: Molecular docking of hyperoside, rutin, and chlorogenic acid with BTB domain of Keap1, Figure S15: Effect of drug interactions between chlorogenic acid and isoquercitrin or quercetin on the Glu-induced HT22 cell death, Figure S16: Hypothetical model of the protective and synergistic effects of isoquercitrin and quercetin in DMLE on the Glu-induced oxidative cell death in HT22 cells, Table S1: 1H-NMR and 13C-NMR chemical shift of isolated compounds, Table S2: Range, linearity, limit of detection (LOD), and limit of quantitation (LOQ) of HPLC-UV assay, Table S3: Precision and accuracy of HPLC-UV assay.
