# Supplementary material for: Synergistic Protection by Isoquercitrin and Quercetin against Glutamate-Induced Oxidative Cell Death in HT22 Cells via Activating Nrf2 and HO-1 Signaling Pathway: Neuroprotective Principles and Mechanisms of Dendropanax morbifera Leaves
Source: Antioxidants (Basel). 2021 Apr 2;10(4):554. doi: 10.3390/antiox10040554 (PMC8066007; doi:10.3390/antiox10040554)
Supplement: Supplementary file 1 [file antioxidants-10-00554-s001.pdf]

Supplementary Materials

Synergistic Protection by Isoquercitrin and Quercetin against Glutamate-Induced Oxidative Cell Death in HT22 Cells Via Activating Nrf2 and HO-1 Signaling Pathway: Neuroprotective Principles and Mechanisms of *Dendropanax morbifera* Leaves

Hye-Jin Park [1], Ha-Neul Kim [1,2], Chul Young Kim [3], Min-Duk Seo [1,2,*] and Seung-Hoon Baek [1,*]

[1] College of Pharmacy and Research Institute of Pharmaceutical Science and Technology (RIPST), Ajou University, Suwon 16499, Republic of Korea; hyejin133@ajou.ac.kr (H-J. P.), skylive02@ajou.ac.kr (H-N. K.); mdseo@ajou.ac.kr (M-D. S.), and shbaek@ajou.ac.kr (S-H. B.)

[2] Department of Molecular Science and Technology, Ajou University, Suwon 16499, Republic of Korea

[3] College of Pharmacy and Institute of Pharmaceutical Science and Technology, Hanyang University, Ansan-si 15588, Republic of Korea; chulykim@hanyang.ac.kr (C.Y. K.)

* Correspondence: mdseo@ajou.ac.kr (M.-D. S.); shbaek@ajou.ac.kr (S.-H. B.)

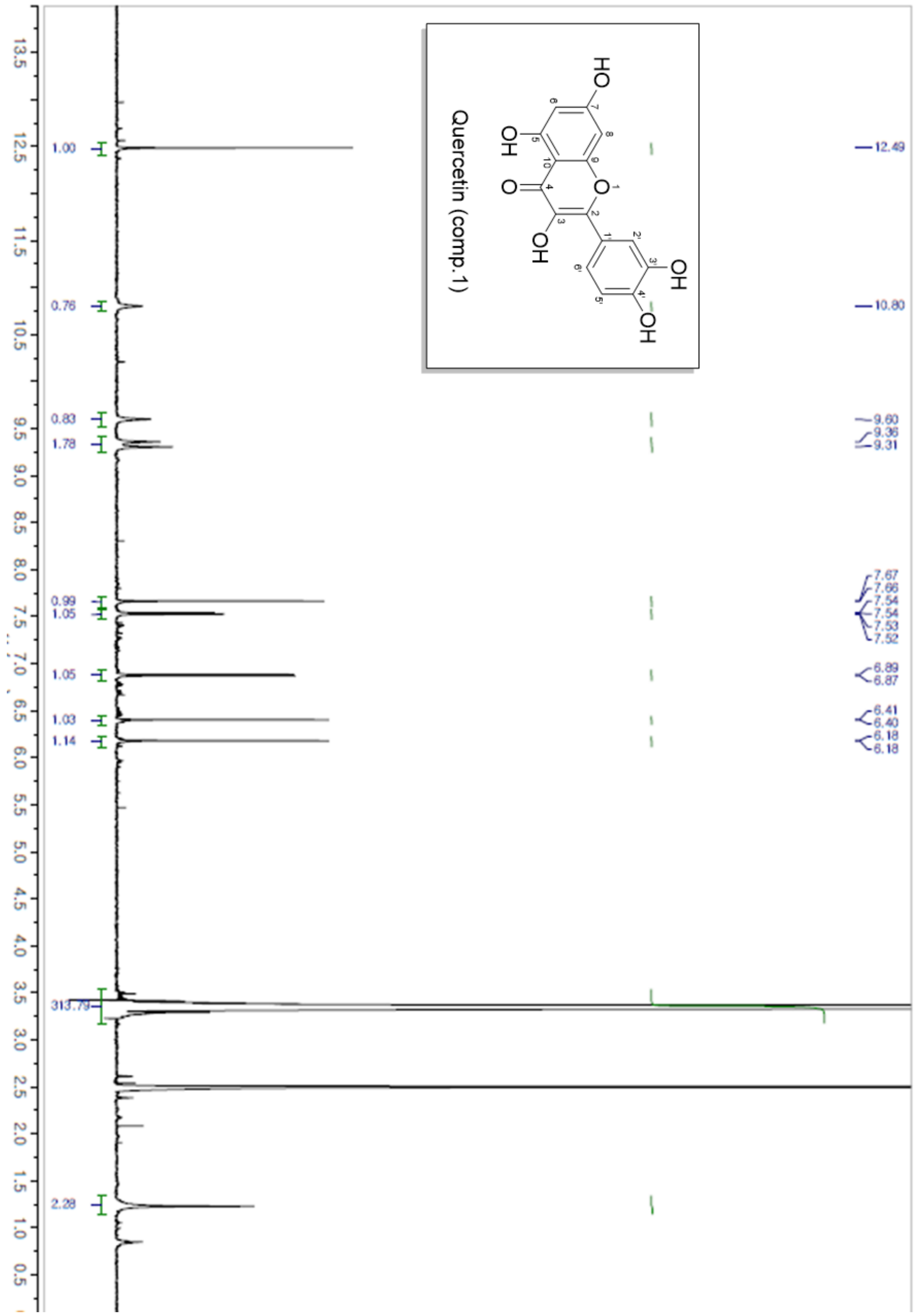

Figure S1. ^{1}H-NMR spectrum of compound 1 (quercetin, 500 MHz, DMSO-d_6).

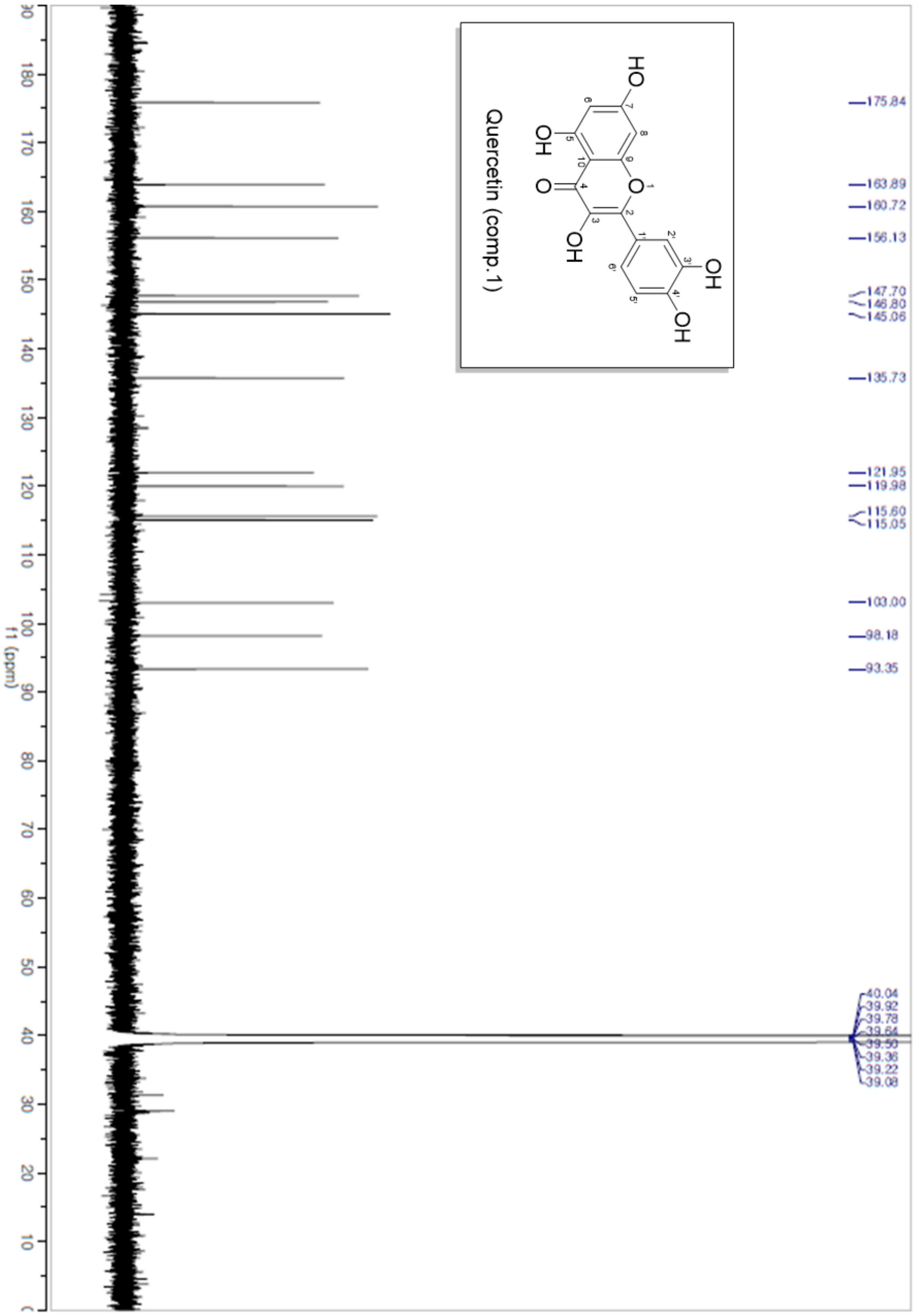

Figure S2. ^{13}C-NMR spectrum of compound 1 (quercetin, 125 MHz, DMSO-d_6).

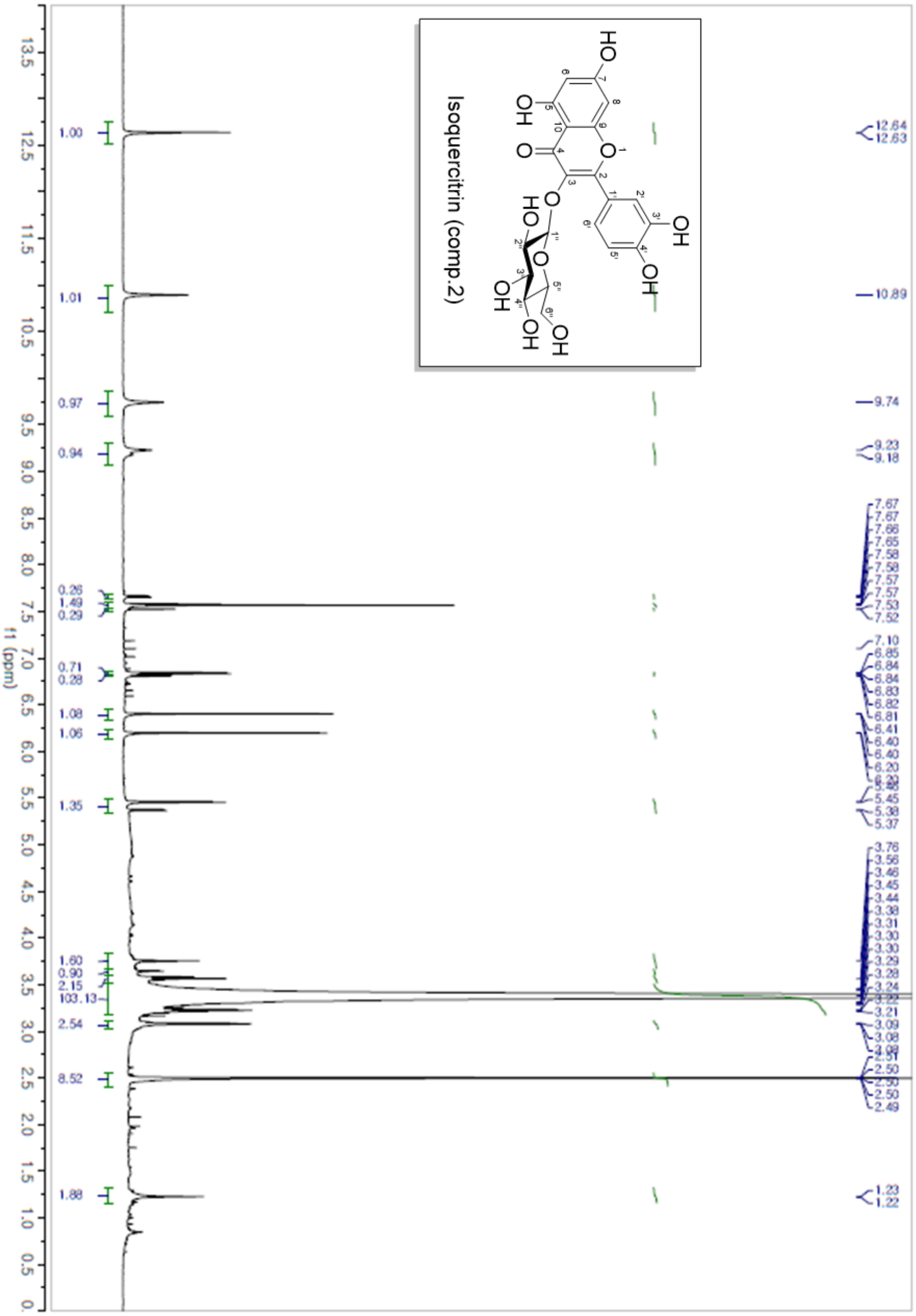

Figure S3. ^{1}H-NMR spectrum of compound 2 (isoquercitrin, 500 MHz, DMSO-d_6).

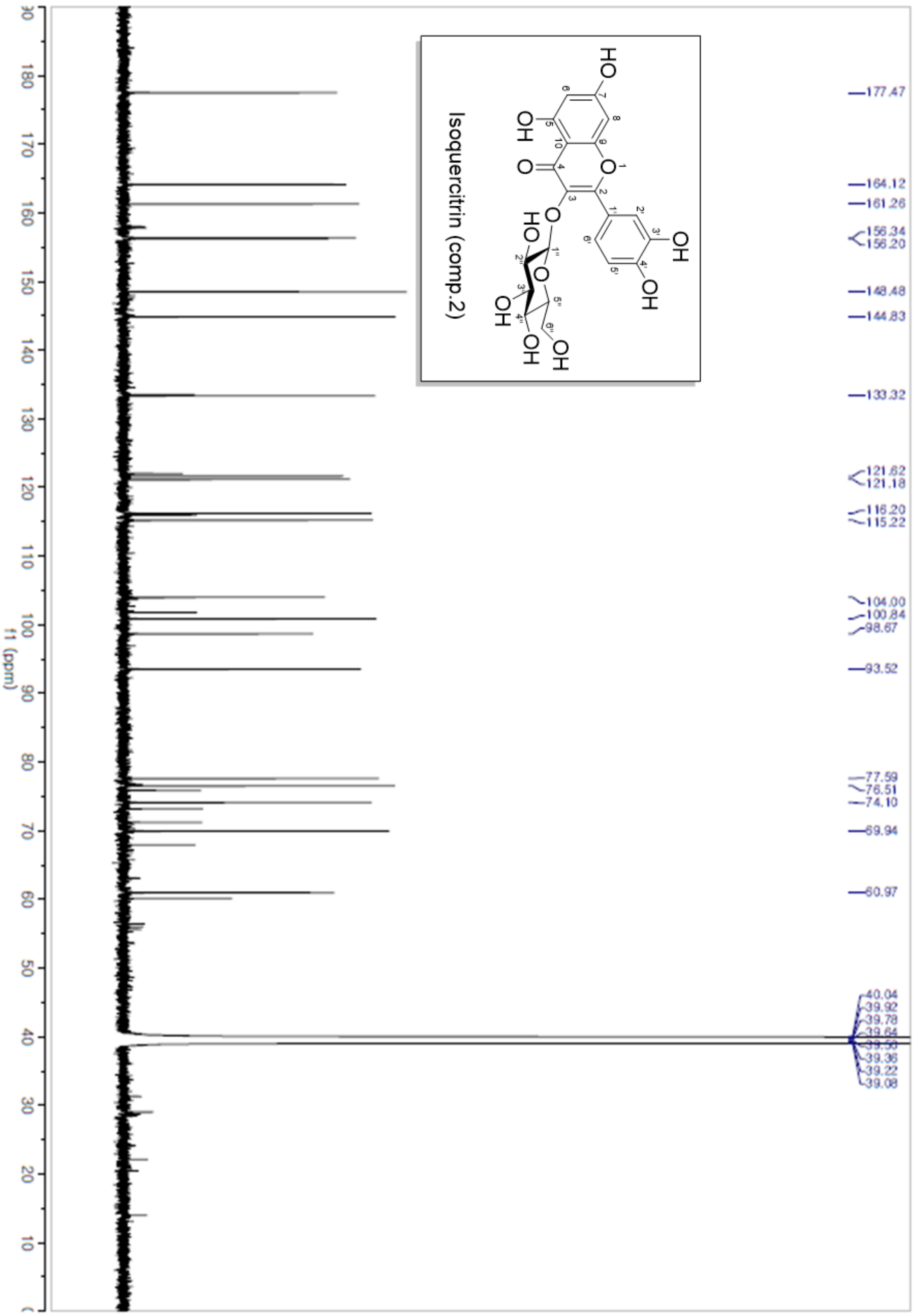

Figure S4. ^{13}C-NMR spectrum of compound 2 (isoquercitrin, 125 MHz, DMSO-d_6).

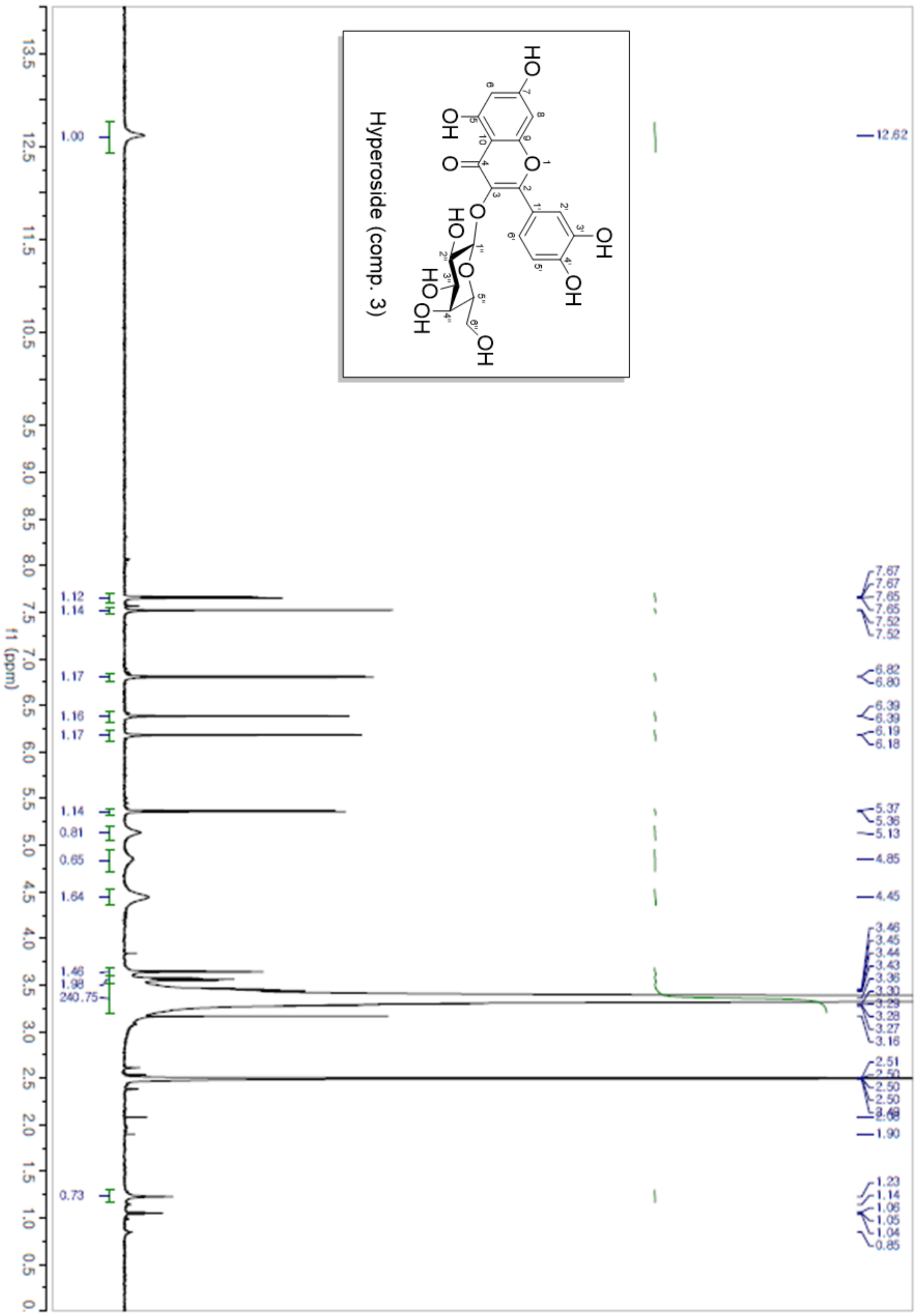

Figure S5. ^{1}H-NMR spectrum of compound 3 (hyperoside, 500 MHz, DMSO-d_6).

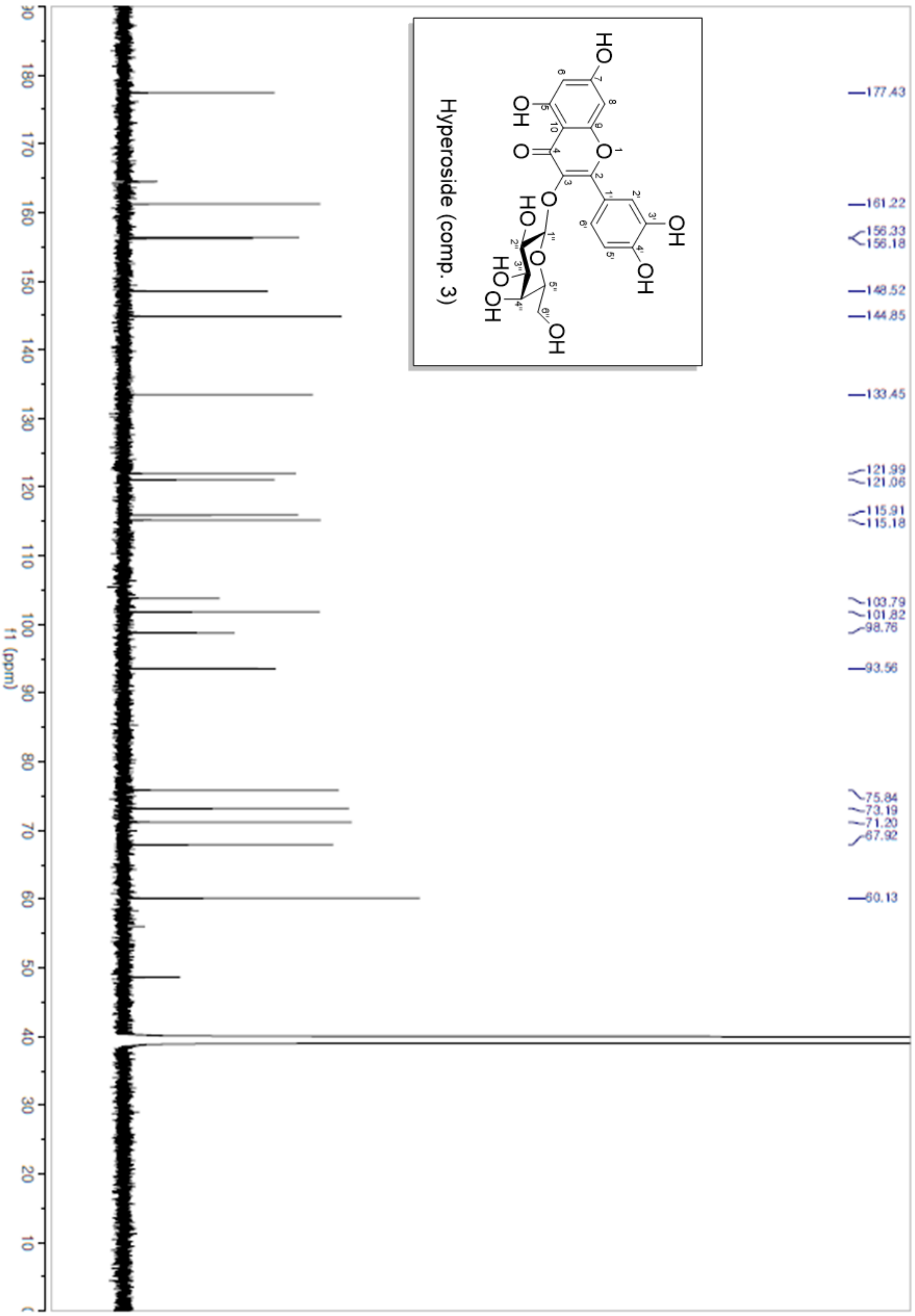

Figure S6. ^{13}C-NMR spectrum of compound 3 (hyperoside, 125 MHz, DMSO-d_6).

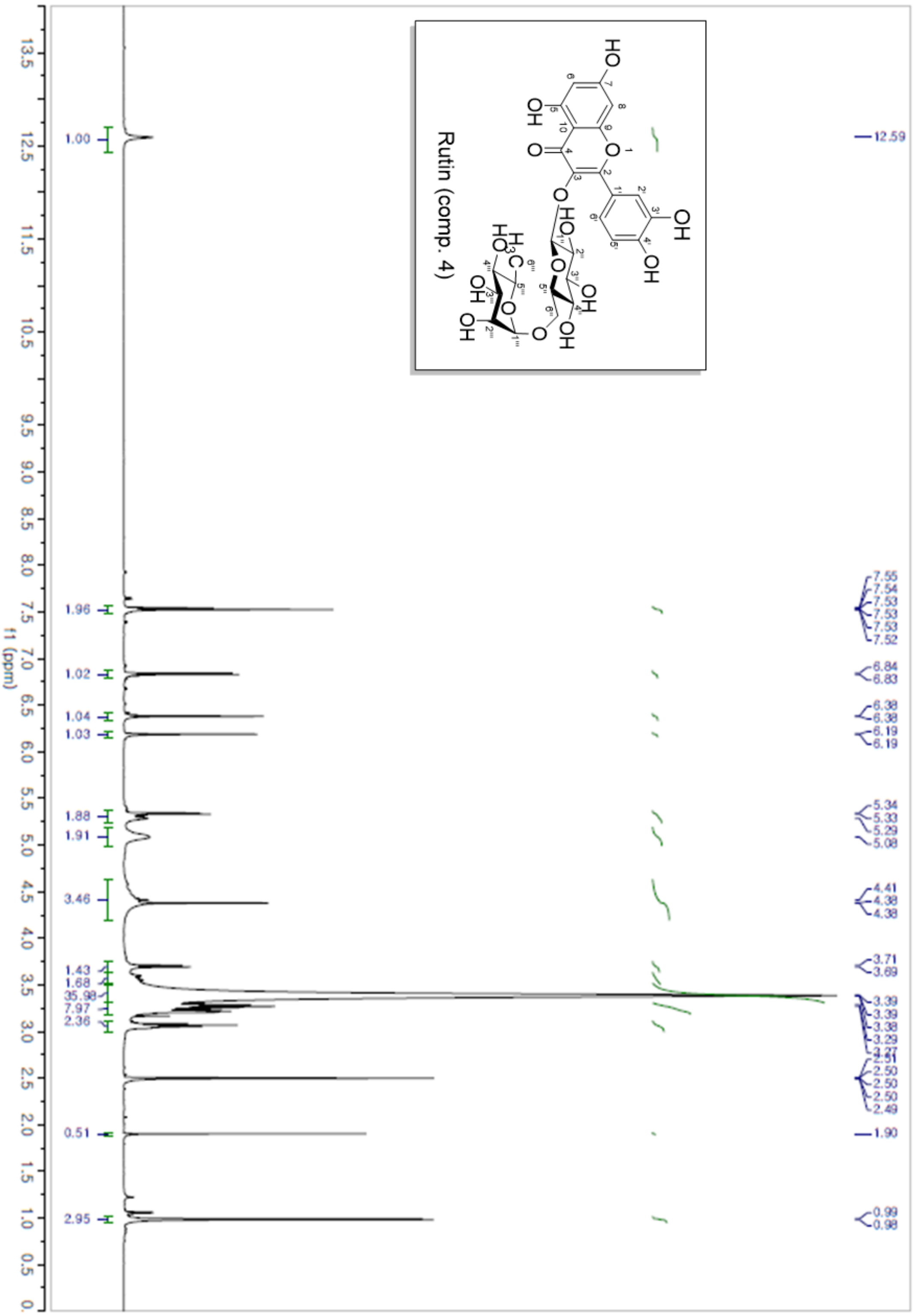

Figure S7. ^{1}H-NMR spectrum of compound 4 (rutin, 500 MHz, DMSO-d_6).

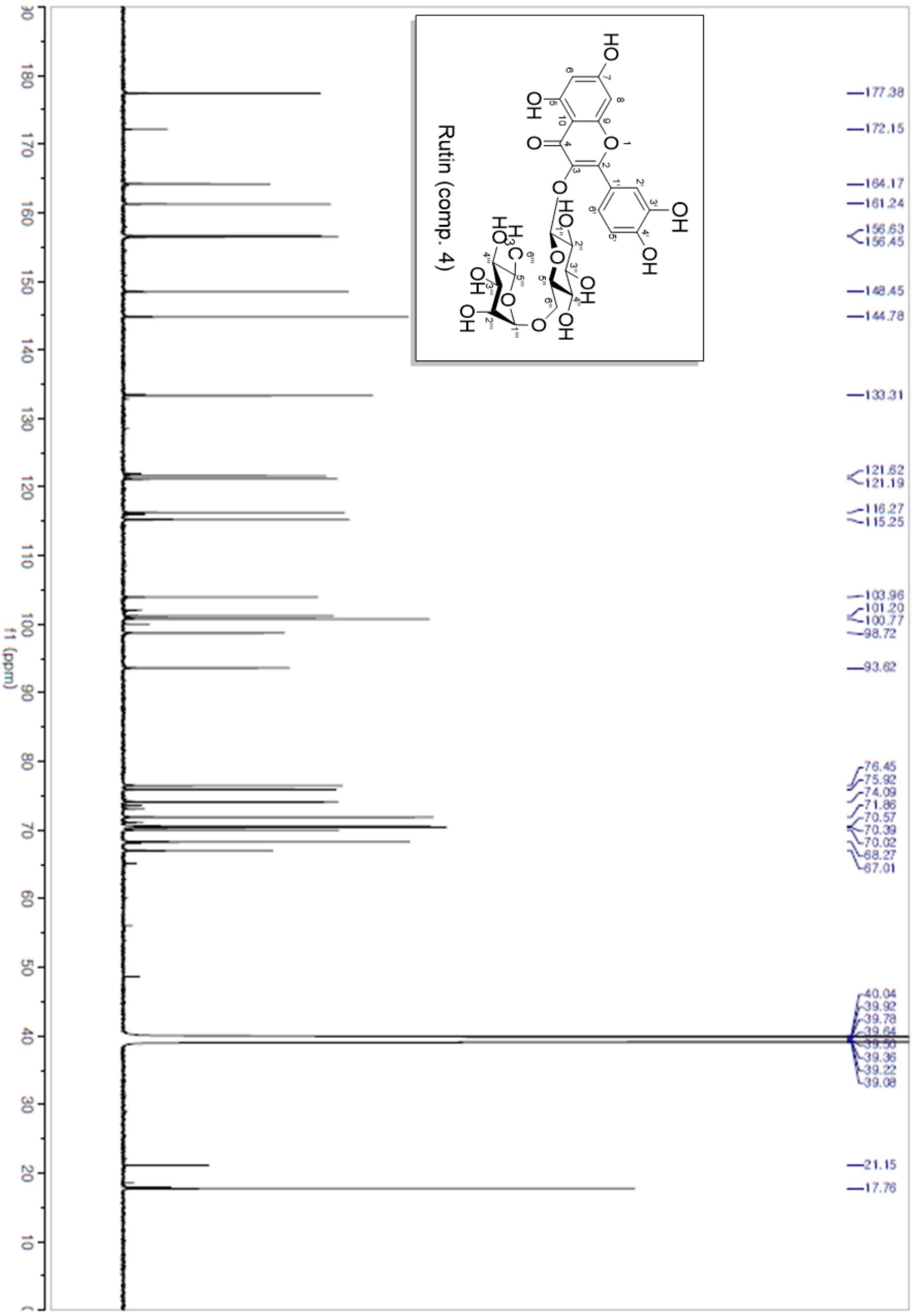

Figure S8. ^{13}C-NMR spectrum of compound 4 (rutin, 125 MHz, DMSO-d_6).

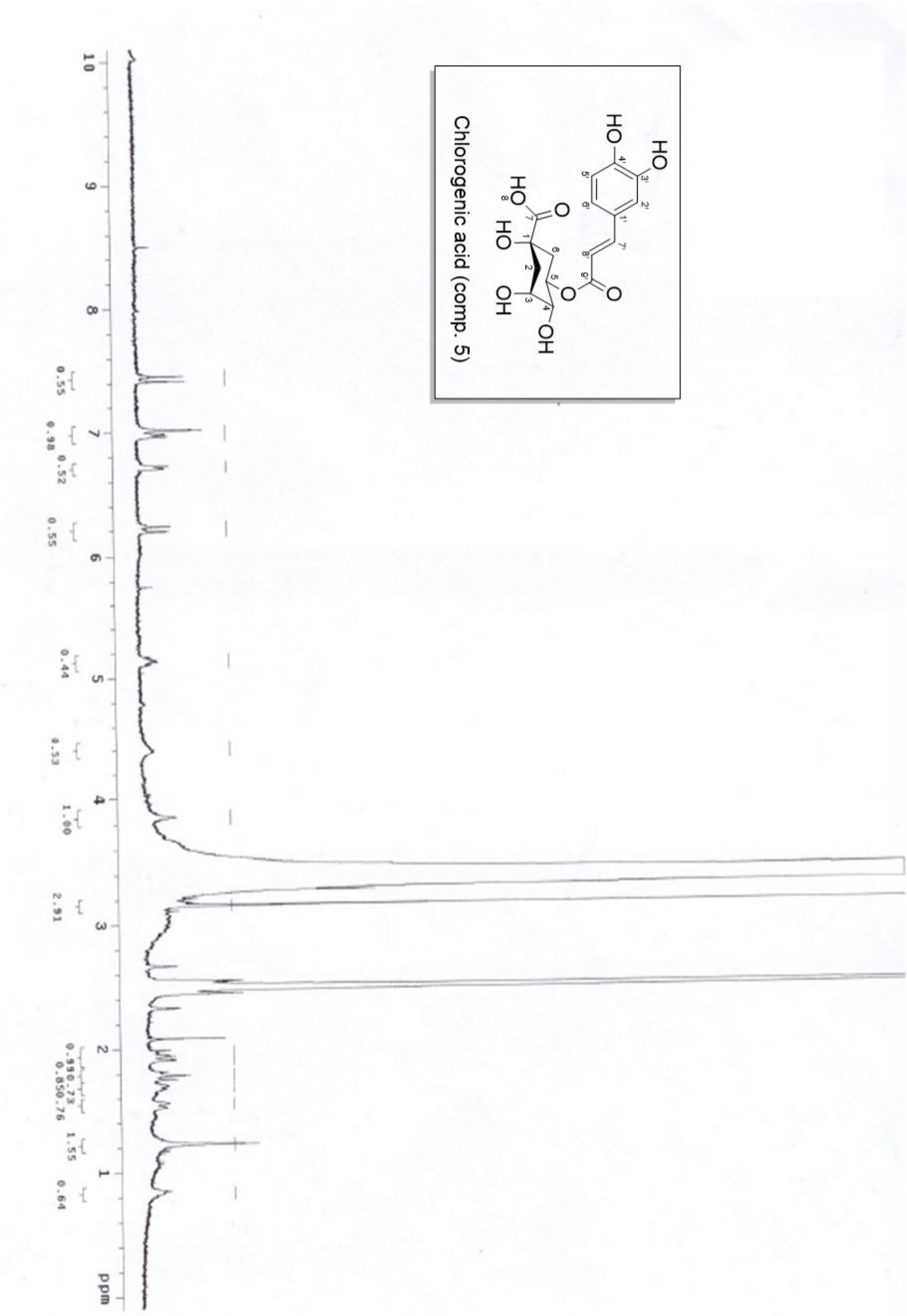

Figure S9. ^{1}H-NMR spectrum of compound 5 (chlorogenic acid, 400 MHz, DMSO-d_6).

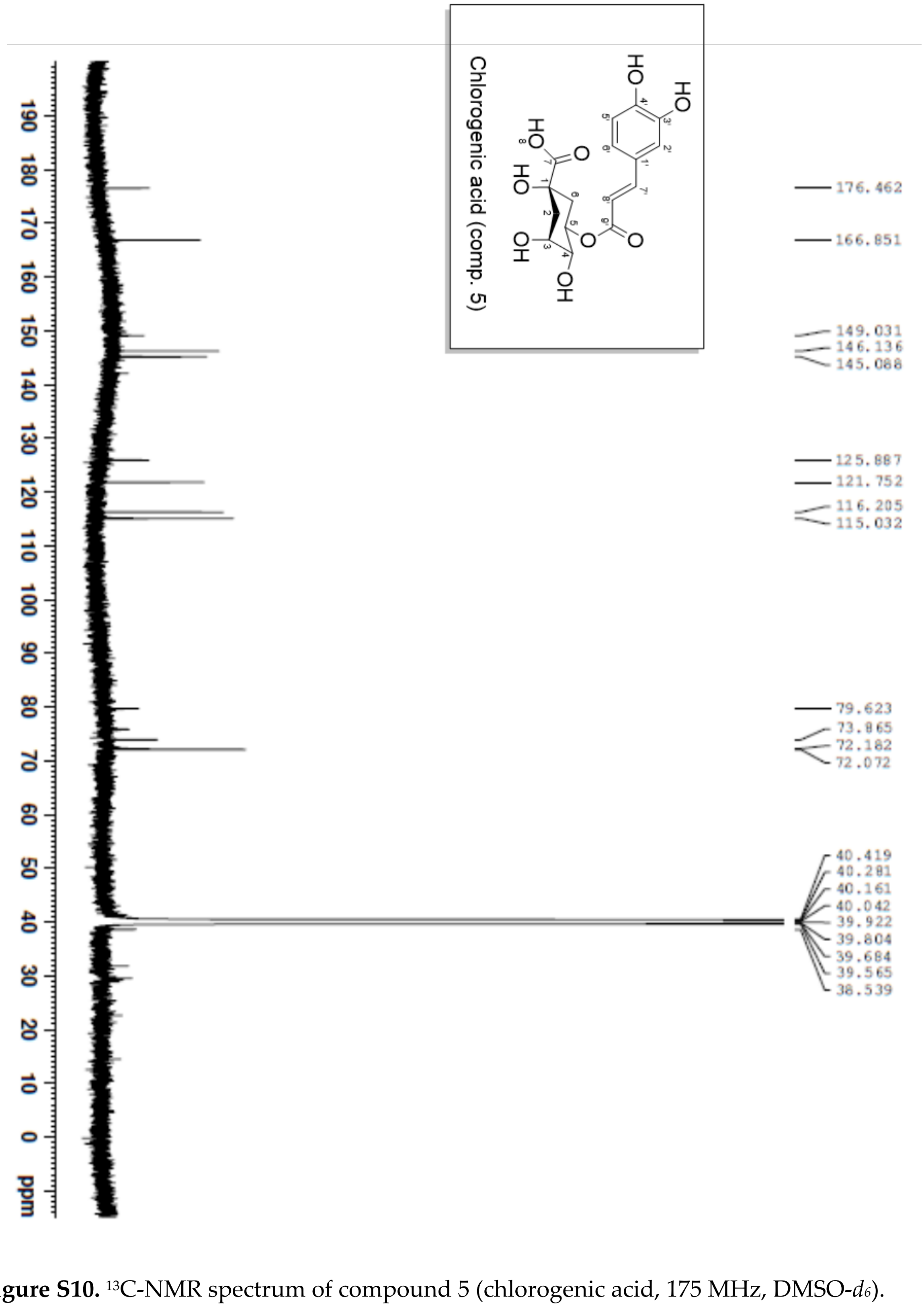

Figure S10. ^{13}C-NMR spectrum of compound 5 (chlorogenic acid, 175 MHz, DMSO-d_6).

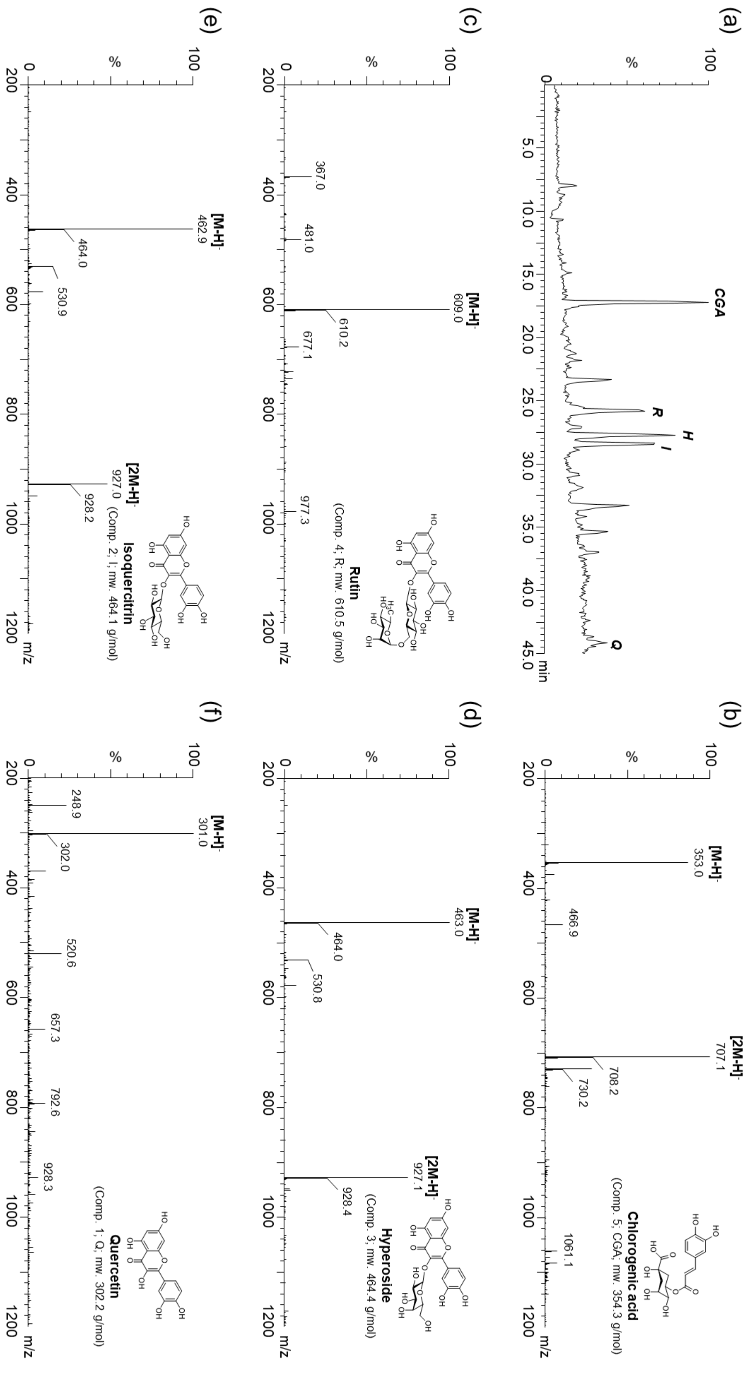

Figure S11. LC-ESI-MS total ion chromatogram of DMLE and mass spectra of isolated compounds. (**a**) Total ion chromatogram of DMLE (1000 μg/mL) using LC-ESI-MS negative ion mode; Mass spectra of chlorogenic acid (**b**), rutin (**c**), hyperoside (**d**), isoquercitrin (**e**), and quercetin (**f**); DMLE (1000 μg/mL) was analyzed using LC-ESI/MS on negative acquisition mode (Micromass ZQ, Waters, Milford, MA, USA) at the following analytical conditions; injection volume, 10 μL; flow rate, 0.5 mL/min; mobile phase A, acetonitrile; mobile phase B, 0.1% formic acid; solvent gradient program, 10% A (0 min), 16% (4 min), 19% (10 min), 22% (20 min), and 50% (45 min); CGA, chlorogenic acid; H, hyperoside; I, isoquercitrin; Q, quercetin; R, rutin; DMLE, ethyl acetate soluble extracts of *Dendropanax morbifera* leaves

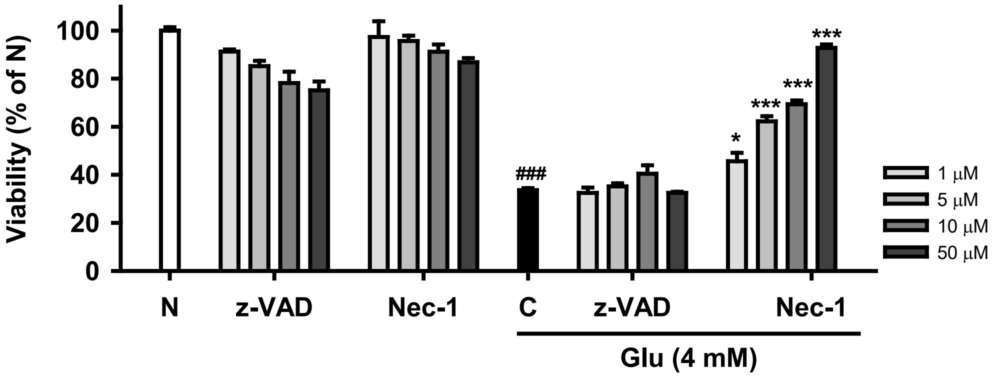

Figure S12. Effect of z-VAD-fmk and Necrostatin-1 on the Glu-induced HT22 cell death. Cells were treated with compounds and/or Glu for 24 h. Cell viability was assessed using MTT assay. Significant differences were determined using Student's *t*-test (### $P < 0.001$, vs. N) or One-way ANOVA followed by Tukey's multiple comparison test (* $P < 0.05$, *** $P < 0.001$, vs. C); Glu, glutamate; N, non-treated normal cell; C, control cells treated with Glu only; z-VAD, z-VAD-fmk; Nec-1, Necrostatin-1.

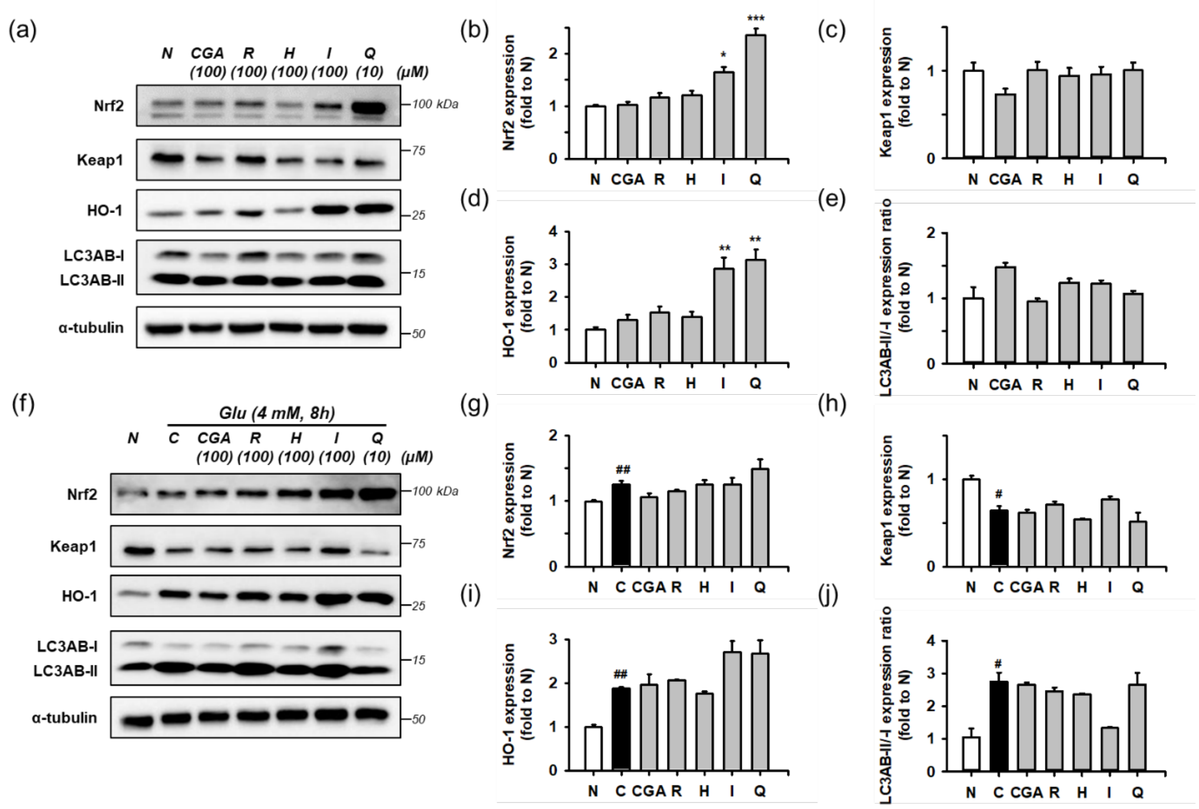

Figure S13. Effect of isolated compounds on the Nrf2/HO-1 and autophagy pathways. (a) Representative blot images showing the effect of compounds without Glu treatment; (**b-e**) Quantitative data of protein levels of Nrf2, Keap1, HO-1, and ratio of LC3AB-II/-I in blot (a); (**f**) Representative blot images showing the effect of compounds with Glu treatment; (**g-j**) Quantitative data of protein levels of Nrf2, Keap1, HO-1, and ratio of LC3AB-II/-I in blot (f); After HT22 cells were incubated with compound or Glu for 8 h, cell lysates were analyzed using western blotting; Significant differences were determined using Student's *t*-test ($^{\#}$ $P < 0.05$, $^{\#\#}$ $P < 0.01$, vs. N) or One-way ANOVA followed by Tukey's multiple comparison test (* $P < 0.05$, ** $P < 0.01$, *** $P < 0.001$, vs. C); Glu, glutamate; N, non-treated normal cell; C, control cells treated with Glu only; CGA, chlorogenic acid; H, hyperoside; I, isoquercitrin; Q, quercetin; R, rutin.

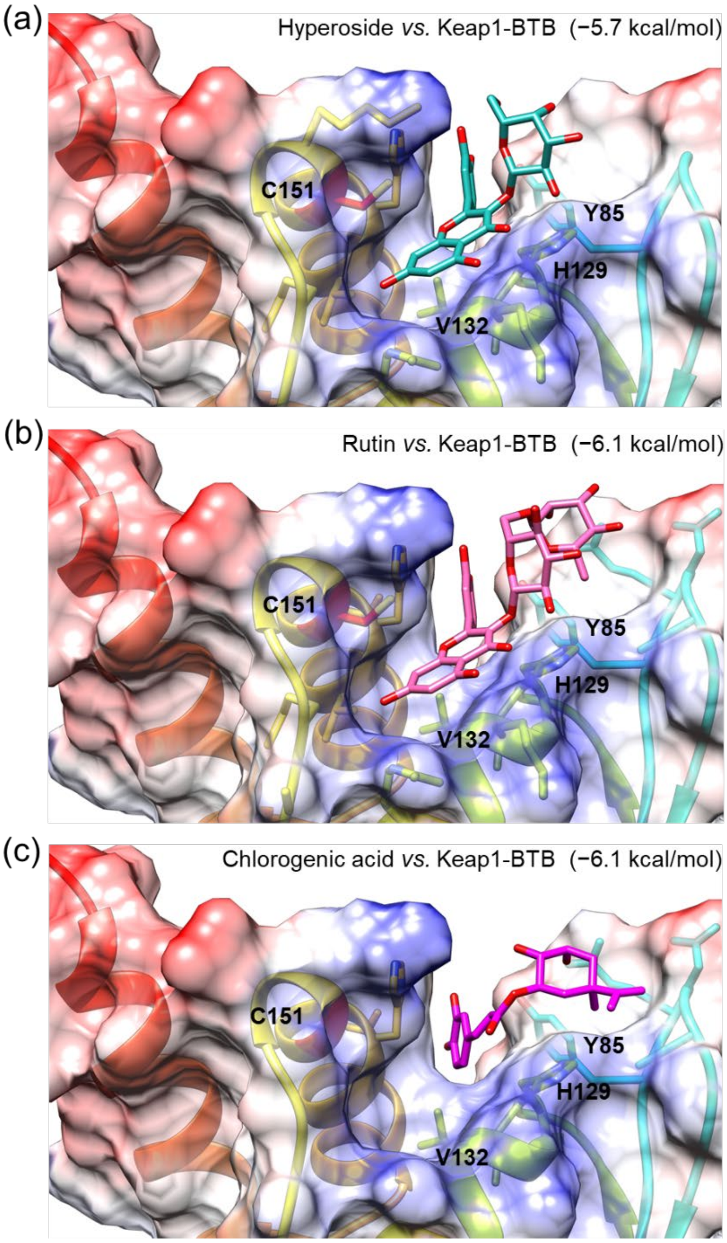

Figure S14. Molecular docking of hyperoside, rutin, and chlorogenic acid with BTB domain of Keap1. (**a**) Hyperoside; (**b**) Rutin; (**c**) Chlorogenic acid; The binding pocket of Keap1-BTB is represented as surface presentations. Chemical bonds of hyperoside, rutin, and chlorogenic acid are depicted as cyan, light pink, and magenta sticks, respectively. The residues with negatively and positively charged residues are colored red and blue, respectively; Keap1-BTB, BTB domain of Keap1.

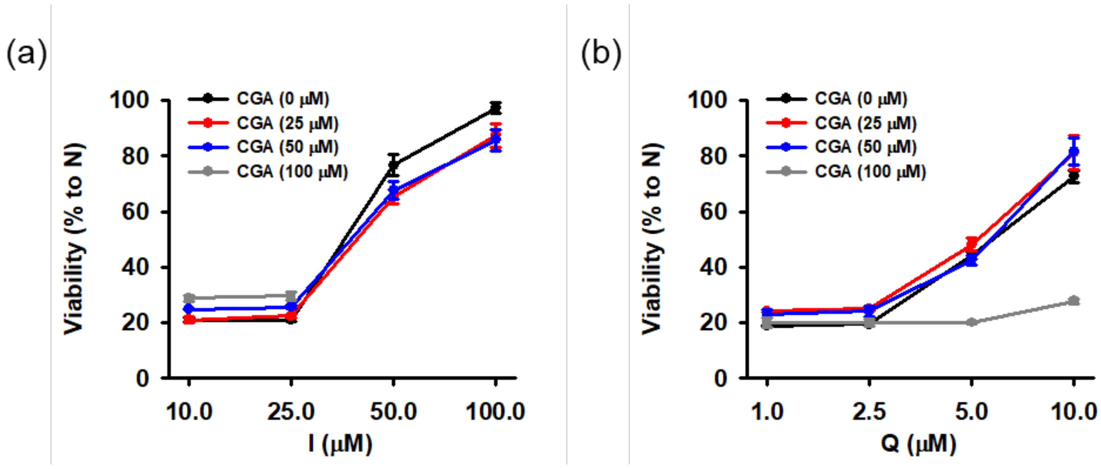

Figure S15. Effect of drug interactions between chlorogenic acid and isoquercitrin or quercetin on the Glu-induced HT22 cell death. (**a**) Effect of drug interaction between I and CGA; (**b**) Effect of drug interaction between Q and CGA; HT22 cells were treated I or Q alone or combination with CGA in the presence of 4 mM Glu. After incubation for 24 h, cell viability was determined using MTT assay; Glu, glutamate; CGA, chlorogenic acid; I, isoquercitrin; Q, quercetin.

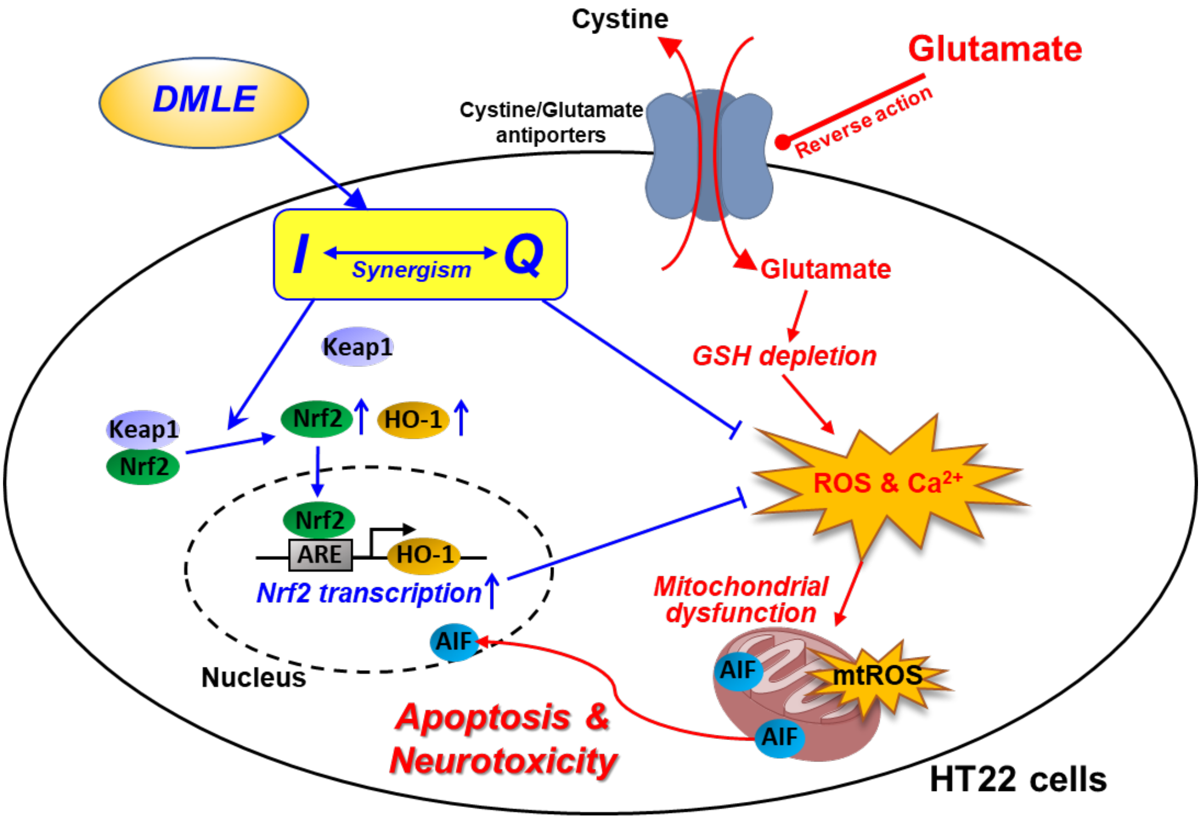

Figure S16. Hypothetical model of the protective and synergistic effects of isoquercitrin and quercetin in DMLE on the Glu-induced oxidative cell death in HT22 cells. Red line and text indicated the toxicity of glutamate. Blue line and text indicated the protective effects of isoquercitrin and quercetin against glutamate toxicity; DMLE, ethyl acetate-soluble extracts of *Dendropanax morbifera* leaves; I, isoquercitrin; Q, quercetin.

Table S1. Summary of ^{1}H-NMR and ^{13}C-NMR spectroscopic data of isolated compounds. (*J* in Hz)

Position	Comp. 1 (Quercetin)		Comp. 2 (Isoquercitrin)		Comp. 3 (Hyperoside)		Comp. 4 (Rutin)		Comp. 5 (Chlorogenic acid)	
	δ_H	δ_C	δ_H	δ_C	δ_H	δ_C	δ_H	δ_C	δ_H	δ_C
**1**										79.62
**2**		147.70		156.34		158.33		156.63		38.54
**3**	9.60 (1H, s, HO-3)	135.73		133.32		133.45		133.31	3.86 (1H, br s, H-3)	72.07
**4**		175.84		177.47		177.43		177.38	3.40 (1H, br d, *J* = 6.8, H-4)	73.87
**5**	12.49 (1H, s, HO-5)	156.13	12.64 (1H, s, HO-5)	161.26	12.62 (1H, s, HO-5)	161.22	12.59 (1H, s, H-5)	161.24	5.16 (1H, br d, *J* = 6.8, H-5)	72.16
**6**	6.18 (1H, d, *J* = 1.8, H-6)	98.18	6.20 (1H, d, *J* = 1.98, H-6)	98.67	6.19 (1H, d, *J* = 1.9, H-6)	98.78	6.19 (1H, d, *J* = 1.9, H-6)	98.72	1.93-2.11 (2H, m, H-6)	40.42
**7**	10.80 (1H, s, HO-7)	163.89	10.89 (1H, s, HO-7)	164.12				164.17		176.46
**8**	6.41 (1H, d, *J* = 1.8, H-8)	93.35	6.41 (1H, d, *J* = 2.19, H-8)	93.52	6.41 (1H, d, *J* = 1.9, H-8)	93.56	6.38 (1H, d, *J* = 1.9, H-8)	93.62		
**9**		160.72		156.20		158.18		156.45		
**10**		103.00		104.00		103.79		100.77		
**1'**		121.95		121.18		121.06		121.19		125.89
**2'**	7.67 (1H, d, *J* = 1.2, H-2')	115.60	7.67 (1H, d, *J* = 2.4, H-2')	116.20	7.67 (1H, d, *J* = 2.1, H-2')	115.18		115.27	7.01 (1H, d, *J* = 2.0, H-2′)	115.03
**3'**		145.08	9.18 (1H, s, HO-3')	144.83		144.85		144.78		145.09
**4'**	9.31 (1H, s, HO-4')	146.80	9.74 (1H, s, HO-4')	148.48		148.52		148.45		149.03
**5'**	6.87 (1H, d, *J* = 8.4, H-5')	115.05	6.85 (1H, d, *J* = 8.5, H-5')	115.22	6.82 (1H, d, *J* = 8.0, H-5')	115.91	6.84 (1H, d, *J* = 8.75, H-5')	116.27	6.72 (1H, d, *J* = 8.2, H-5′)	116.21
**6'**	7.53 (1H, d, *J* = 8.1, H-6')	119.98	7.52 (1H, dd, *J* = 2.2, 8.5, H-6')	121.62	7.52 (1H, dd, *J* = 2.1, 8.0, H-6')	121.99	7.55, 7.52 (2H, d, *J* = 1.9, H-2', 6')	121.62	6.98 (1H, dd, *J* = 2.0, 8.2, H-6′)	121.75
**7'**									7.45 (1H, d, *J* = 15.9, H-7′)	146.14
**8'**									6.23 (1H, d, *J* = 15.9, H-8′)	115.41
**9'**										166.85
**1''**			5.38 (1H, d, *J* = 7.76, H-1'')	100.84	5.37 (1H, d, *J* = 7.7, H-1'')	101.82	5.34 (1H, d, *J* = 7.8, H-1'')	101.20		
**2''**			3.09 ~3.76 (6H, H-2''~H-6'')	74.10	3.58 (1H, dd, *J* = 8.0, 9.3, H-2'')	71.20		74.09		
**3''**				76.51		73.19		75.92		
**4''**				69.94	3.65 (1H, d, *J* = 3.2, H-4'')	67.92		70.02		
**5''**				77.59		75.84		76.45		
**6''**				60.97	3.55 (1H, dd, *J* = 5.6, 10.1, H-6'')	60.13	4.38 (1H, s, H-1''')	67.01		
**1'''**							3.39 (1H, d, *J* = 8.8, H-2''')	103.96		
**2'''**								70.57		
**3'''**								70.39		
**4'''**								71.88		
**5'''**								68.27		
**6'''**							0.99 (3H, d, *J* = 6.8, H-6''')	17.76		

Abbreviations: s, singlet; d, doublet; dd, double doublet; br, broad; m, multiplet.

Table S2. Range, linearity, limit of detection (LOD), and limit of quantitation (LOQ) of HPLC-UV assay.

Analyte	Range (μg/mL)	Linearity		LOD (μg/mL)	LOQ (μg/mL)
		Regression equation	R^2		
Chlorogenic acid	1 ~ 10	y = 10.583x – 0.4637	0.9997	0.05	0.13
Rutin	1 ~ 10	y = 20.404x - 1.0546	0.9999	0.01	0.05
Hyperoside	1 ~ 10	y = 40.91x - 3.0238	0.9998	0.01	0.04
Isoquercitrin	1 ~ 10	y = 25.149x - 1.3224	0.9999	0.02	0.05
Quercetin	0.1 ~ 1	y = 29.577x - 1.0131	0.9995	0.01	0.02

Table S3. Precision and accuracy of HPLC-UV assay.

Sample	Analyte	Spiked (μg/mL)	Intra-day (%)		Inter-day (%)	
			Precision[1)]	Accuracy[2)]	Precision[1)]	Accuracy[2)]
DMLE	Chlorogenic acid	1	4.9	130.1 ± 6.4	4.0	133.2 ± 5.4
		5	2.3	100.6 ± 2.3	1.4	114.9 ± 1.6
		10	1.6	101.5 ± 1.6	1.9	108.9 ± 2.1
	Rutin	1	1.8	97.4 ± 1.7	2.8	99.5 ± 2.8
		5	0.8	100.5 ± 0.8	0.2	100.7 ± 0.2
		10	1.3	99.7 ± 1.3	0.8	100.2 ± 0.8
	Hyperoside	1	1.8	100.9 ± 1.8	2.9	102.1 ± 2.9
		5	0.6	100.3 ± 0.6	0.9	100.5 ± 0.9
		10	0.3	100.3 ± 0.3	1.9	100.3 ± 1.9
	Isoquercitrin	1	1.9	98.9 ± 1.9	4.4	102.0 ± 4.5
		5	0.4	99.7 ± 0.4	0.7	100.2 ± 0.7
		10	0.2	100.8 ± 0.2	0.6	100.5 ± 0.6
	Quercetin	0.1	2.7	97.0 ± 2.7	8.3	101.9 ± 8.5
		0.5	3.1	102.8 ± 3.2	1.2	101.4 ± 1.3
		1	0.5	100.5 ± 0.5	1.5	100.3 ± 1.5
DMB	Rutin	1	2.5	104.3 ± 2.6	0.9	105.0 ± 1.0
		5	0.4	100.8 ± 0.4	0.5	100.6 ± 0.5
		10	0.4	100.5 ± 0.4	0.3	100.7 ± 0.3

[1)] Precision was expressed as relative standard deviation (RSD %)

[2)] Accuracy was expressed as mean ± standard deviation of recovery (%)
